# Species diffusion in clinopyroxene solid solution in the diopside–anorthite system

**DOI:** 10.1007/s00410-019-1571-9

**Published:** 2019-05-13

**Authors:** Matthias Bernhard Lierenfeld, Xin Zhong, Eric Reusser, Karsten Kunze, Benita Putlitz, Peter Ulmer

**Affiliations:** 1 Institute of Geochemistry and Petrology, Swiss Federal Institute of Technology (ETH Zürich), Clausiusstrasse 25, 8092 Zurich, Switzerland; 2 Department of Geosciences, Physics of Geological Processes, The Njord Center, University of Oslo, PO Box 1048, Blindern, 0371 Oslo, Norway; 3 Scientific Center for Optical and Electron Microscopy, Swiss Federal Institute of Technology (ETH Zürich), Auguste-Piccard-Hof 1, 8093 Zurich, Switzerland; 4 Institut des Sciences de la Terre, Université de Lausanne, Mouline-Géopolis, 1015 Lausanne, Switzerland

**Keywords:** Multicomponent coupled diffusion, Eigenvalues, Eigenvectors, Pyroxene diffusion, Seed overgrowth technique

## Abstract

The coupled multicomponent diffusion of the species Ca_2_Si_2_O_6_, CaAl_2_SiO_6_ and Mg_2_Si_2_O_6_ was determined in diopside crystals in the diopside/anorthite (Di/An) system at temperatures (*T*) of 1110–1260 °C and oxygen fugacities (*fO*_*2*_) between 1.0 log unit below and above the fayalite–magnetite–quartz equilibrium (FMQ ± 1). Diffusion couples were prepared by the seed overgrowth technique. Element concentration profiles were measured perpendicular to the rim/core interface by step-scanning profiling with a field emission gun scanning electron microscope (FEG-SEM). The multicomponent diffusion matrix was solved by fitting its eigenvalues (*λ*) and eigenvectors (*v*) to the measured concentration profiles. The full diffusion matrix D can be recovered by using the formula $D = P\varLambda P^{ - 1}$ resulting in the following equation:

$$D_{\text{Di/An}} = \left[ {\begin{array}{*{20}c} {1.00} & { - 0.67} \\ { - 0.38} & {1.00} \\ \end{array} } \right]\left[ {\begin{array}{*{20}c} {\lambda 1\left( T \right)} & 0 \\ 0 & {\lambda 2\left( T \right)} \\ \end{array} } \right]\left[ {\begin{array}{*{20}c} {1.00} & { - 0.67} \\ { - 0.38} & {1.00} \\ \end{array} } \right]^{ - 1} .$$

The eigenvalues (*λ1* and *λ2*) represent upper limit values and are described by the following Arrhenius-type equations:

$$\lambda 1 \left( {\text{Di/An}} \right) = 10^{ - 15.98 \pm 1.17} \times \exp \left[ {\frac{{ - 114.4 \pm 32.8 {\text{kJ/mol}}}}{RT}} \right],$$

$$\lambda 2 \left( {\text{Di/An}} \right) = 10^{ - 16.23 \pm 1.17} \times { \exp }\left[ {\frac{{ - 114.4 \pm 32.8 {\text{kJ/mol}}}}{RT}} \right],$$

where *λ*1 and *λ*2 are the first and second eigenvalue of the diffusion matrix in m^2^ s^−1^*, R* is the gas constant and *T* is the temperature in *K*. The dominant eigenvalue (*λ1*) is one quarter order of magnitude larger than the second eigenvalue (*λ*2). The eigenvectors are constant for all experiments inferring that the entire D matrix can be described with the eigenvalues as the only *T*-dependent parameter. Additionally, the derived diffusion data and modeling approach were applied to constrain the duration of magmatic processes recorded in zoned clinopyroxene (cpx) phenocrysts from a basaltic, post-plutonic dyke of the Tertiary Adamello batholiths (N-Italy). The results reveal residence times of the overgrown cpx prior to final emplacement in the range of 0.25–1.7 years (lower limit values) testifying that the data and method can be applied to model cpx diffusion profiles in complex natural cpx.

**Electronic supplementary material:**

The online version of this article (10.1007/s00410-019-1571-9) contains supplementary material, which is available to authorized users.

## Introduction

Pyroxenes represent one of the principal ferromagnesian igneous rock-forming minerals being stable over the *T* and *P* conditions of the upper mantle and crust (Deer et al. 2013). Pyroxenes are inosilicates formed by single SiO_3_ chains of connected SiO_4_ tetrahedra with the general formula $M2\left( {R^{2 + } } \right)M1\left( {R^{2 + } } \right)T\left( {2R^{4 + } } \right)O_{6} ,$where M1 is occupied by cations in a regular octahedral coordination (smaller cations such as Mg, Fe^2+^, Fe^3+^ and Al), M2 by cations in a distorted octahedral coordination (larger cations such as Na and Ca), T by tetrahedrally coordinated cations (Si, Al) and R^x+^ is the valence of the metal ions. Due to their chemical diversity (multi-site structure accommodating a significant number of different cations), cpx form various solid solutions in the Ca–Mg–Fe–Al–Na system (Morimoto 1988; Deer et al. 2013). To obtain a more profound understanding of the formation and transformation of such solid solutions, it is important to improve our knowledge regarding diffusion of, e.g., end-member species. In minerals, diffusion is the only solid-state transport mechanism of cations (Zhang 2010) that is responsible for smoothing and/or extinction of mineral zoning patterns produced by mineral overgrowth commonly found in (igneous) minerals such as cpx (Dobosi 1989; Morgan et al. 2004; Lierenfeld and Mattsson 2015). Due to two possible ways such compositionally zoned minerals can be homogenized: (1) changes of the *P*–*T* conditions in the magma reservoir or (2) changing the composition of the magma by rapid differentiation, magma mixing, volatile escape or change in oxidation environment (Sparks et al. 1977; Gerlach and Grove 1982; Blundy et al. 2006; Ginibre et al. 2007). For decades, these mineral zoning patterns have been used as geological archives (Larsen et al. 1938; Tomkeieff 1939). If the diffusion coefficients (D) are known, one can utilize them to obtain geological information about the thermal history recorded by these minerals (Zhang 2010). Originally this was done to determine the cooling rates of extraterrestrial magmas (Wood 1964). Subsequently, applications were extended to terrestrial minerals such as cpx that preserve chemical zoning due to their resistance to internal diffusion modifications (Dimanov and Sautter 2000). For instance, the diffusion of Fe^2+^-Mg in cpx (Müller et al. 2013) is characterized by a lower diffusion rate in comparison to other minerals such as garnet (Borinski et al. 2012), olivine (Dohmen et al. 2007) or spinel (Liermann and Ganguly 2002). These slow diffusion rates make cpx a limiting mineral phase for the determination of the duration of igneous processes (Müller et al. 2013). In the last decades several D values for high Ca–cpx (mainly focusing on self-diffusion and inter-diffusion) were determined by inverse modeling of experimentally produced diffusion profiles (Freer et al. 1982; Brady and McCallister 1983; Sautter et al. 1988; Béjina and Jaoul 1996; Dimanov and Jaoul 1998; Zhang et al. 2010; Müller et al. 2013).

Multicomponent diffusion problems are tackled by scientists for more than half a century (Toor 1964; Cullinan 1965; Gupta and Cooper 1971; Lasaga 1979), however, the motivation for studying the coupled diffusion in diopsidic cpx is driven by the lack of appropriate experiments evaluating the effect of multicomponent coupled diffusion in cpx solid solutions. The coupled diffusive mechanisms can be described by determining the diffusion matrix that couples the element flux to concentration gradients (Liang 2010; Zhang et al. 2010; Claireaux et al. 2016). To calculate the diffusive flux, ideal solid solution mixing is often presumed allowing the substitution of the chemical potential gradient of a component by its concentration gradient which is the quantity typically determined along diffusion profiles (Trial and Spera 1994). Here, the coupled diffusion of the end-member species Ca_2_Si_2_O_6_ (wollastonite), CaAl_2_SiO_6_ (Ca-Tschermak) and Mg_2_Si_2_O_6_ (enstatite) was determined in the Di/An system for diopsides by conducting a series of experiments at ambient *P* in the *T* range 1110 °C–1260 °C and under *fO*_*2*_ conditions ranging from FMQ +1 to FMQ -1. The recovery of the crystal seeds and the quantification of the diffusion profiles were conducted by a combination of several different analytical techniques (X-ray micro-computed tomography (X-ray *µ*CT), electron backscatter diffraction (EBDS) and energy-dispersive X-ray spectroscopy (EDS) attached to a FEG-SEM). The modeling approach is based on the decomposition of the D matrix into its eigenvalues and eigenvectors. This approach was previously employed for the determination of D values in silicate melts (Trial and Spera 1994; Chakraborty et al. 1995a, b; Claireaux et al. 2016) but was so far not applied to coupled diffusion in minerals.

## Methods

### Starting material and diopside crystal seeds

The employed synthetic starting material was composed of 70 *wt.*  *%* diopside and 30 *wt.*  *%* anorthite produced by mixing oxide components in the desired stoichiometric proportions in an agate mortar. The homogenized mixture was fused for 4 h in a Pt crucible at 1400 °C in a box oven in air and quenched in water to produce a homogenous glass, which was re-crushed and -ground to a fine homogenous starting material powder (Table 1).

**Table 1:** Composition of starting material

wt. %	avg. (115)	std.
SiO_2_	51.52	0.16
Al_2_O_3_	10.88	0.16
Fe_2_O_3_	0.25	0.02
MgO	12.62	0.15
CaO	24.47	0.14
Na_2_O	0.01	0.01
Total	99.75	0.22

Numbers in brackets denote number of measurements

*std.* indicates the one sigma standard deviation, *avg.* the average, *Di* refers to diopside, *An* to anorthite

The diopside crystal seeds originate from a dolomite marble of the Adamello massif, Italy. Crystal fragments were polished with 1 *µm* silicon carbide polishing paper to obtain crystal seeds with well-defined crystal surfaces following the visible crystal facets as much as possible. The final crystal seeds were rectangular shaped to mimic the monoclinic crystal symmetry. They varied in size and thickness. Subsequently, they were cleaned in acetone and pre-annealed for 18 h in a CO_2_/H_2_ 1 atmosphere gas-mixing furnace at the designated *fO*_*2*_ conditions of the later experiments at *T* of 1200 °C or 1050 °C depending on the annealing *T* of the following experiment. The pre-annealing was performed to equilibrate possible existing point defect populations in the crystal lattice (Zhang et al. 2010; Cherniak and Liang 2012).

### Experimental setup

**Fig. 1:**
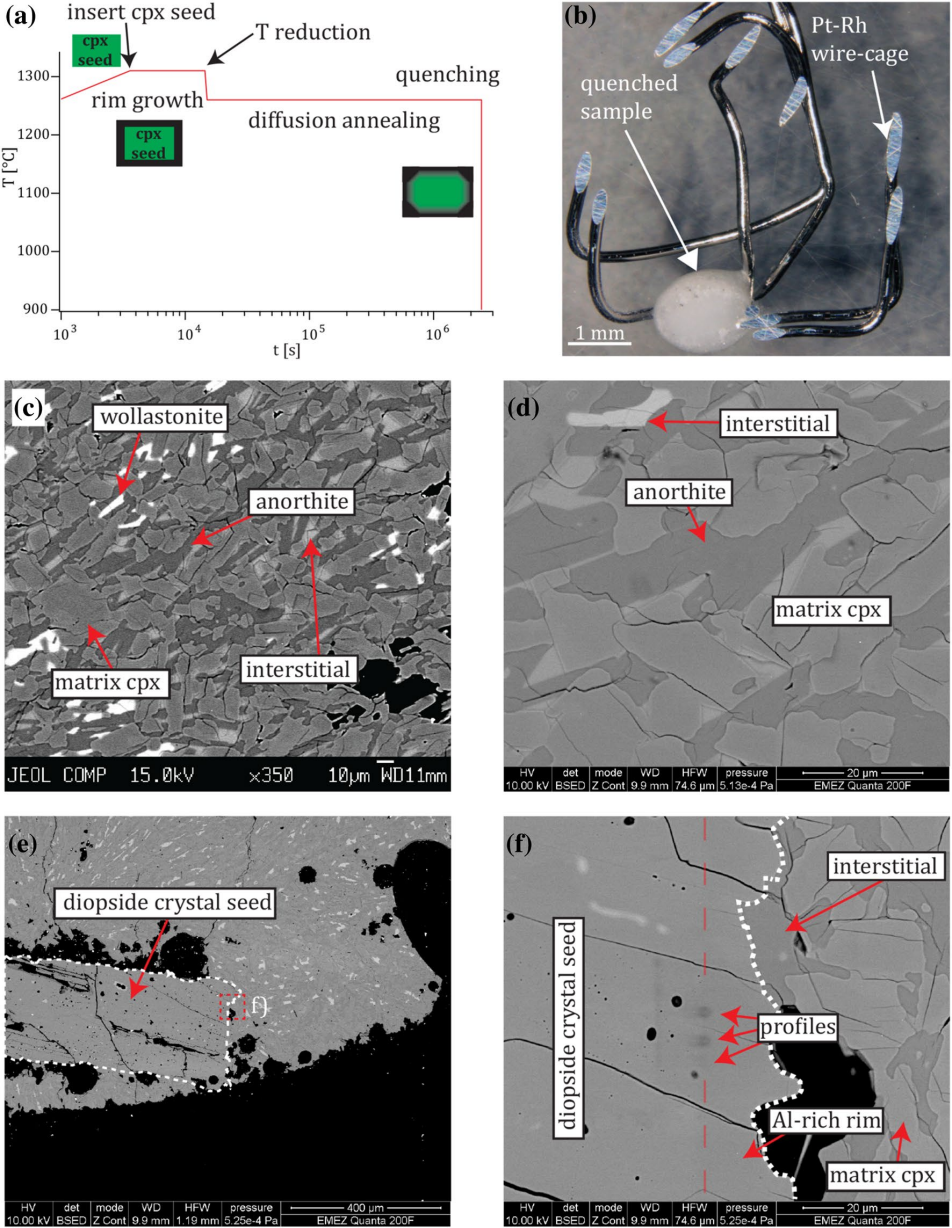
**a** Schematic experimental heating protocol of a 4-week duration experiment. The diopside crystal seed (green rectangle) is surrounded by an overgrowth rim formed within 3 h that is characterized by a step function concentration interface. *T* reduction and long-duration anneal triggers diffusion leading to a measureable diffusion gradient. **b** Experiment after quenching with the Pt–Rh wire-cage containing the sample (fine-grained eutectic mixture and diopside crystal seed). **c**–**f** BSE images at different stages of the measuring procedure (run # 34). **c** Quenched phase assemblage measured by EPMA (without diopside crystal seed). **d** The same anorthite crystal was re-measured and used as standard for Al for the profiling with EDS at the FEG-SEM. **e** The diopside crystal seed used as standard for Si, Ca and Mg embedded in the fine-grained eutectic mixture of (**c**). **f** Close-up illustrating two beam damage remnants of three concentration profiles (one is not visible) measured with EDS at the FEG-SEM across the interface rim/core (red dashed line). Detection of the interface by BSE contrast rendered difficult as the composition of the diopside crystal seed and the rim were too similar for significant differentiation. However, one marker criterion was the appearance of bubbles which exclusively occurred within the seed

To generate diffusion couples and determine coupled diffusion in diopsidic cpx, the seed/overgrowth (SO) technique was employed by rapidly producing an overgrowth rim in epitactic orientation around the seed crystals (orders of magnitude faster than diffusion) followed by a subsequent long period of diffusion anneal (Fig. 1a). This procedure erases the artificially produced overgrowth step interface and generates diffusion profiles (Vielzeuf et al. 2007). The method bases on the assumption that the diffusion profiles can be considered as discontinuous (step function) at the interface of the diffusion couple due to the relatively short overgrowth time in comparison to the following diffusion experiment (Elphick et al. 1985; Ganguly et al. 1998).

A series of experiments (Table 2) was conducted by varying annealing duration (1–4 weeks), *T* (1110–1260 °C) and *fO*_*2*_ conditions (FMQ -1 to FMQ +1). All experiments were exclusively performed in a vertical CO_2_/H_2_ 1 atmosphere gas-mixing furnace, where CO_2_ and H_2_ were mixed in specific ratios to achieve the designated *fO*_*2*_ conditions. For each experiment, a single diopside crystal was engulfed in the finely grained glassy starting material powder. Subsequently, the sample was placed in a Pt–Rh wire-cage acting as sample container and sintered onto the cage for 15 min in a furnace at 1050 °C. The wire-cage (Fig. 1b) was inserted into the upper top part of the gas mixing furnace whose *T* was set at 1310 °C in the central hotspot and after *fO*_*2*_ equilibration, the sample was lowered into the hot zone to produce an Al-enriched cpx rim around the crystal seed by overgrowth (3 h). In the final step, *T* was reduced (300 °C/h) to the designated *T* of the experiment accompanied by an adjustment of the CO_2_/H_2_ ratio corresponding to the same *∆*FMQ value for the diffusion annealing step. Temperatures of the overgrowth steps were selected according to the respective phase diagrams for the diopside–anorthite system (Bowen 1915; Osborn 1942).

**Table 2:** Experimental run conditions of the rim-forming and diffusion experiments

fO_2_	Sample	rim-forming			diffusion annealing		
		*T* (°C)	log *fO*_*2*_	*t* (h)	*T* (°C)	log *fO*_*2*_	*t* (h)
FMQ +1	# 22.2	1310	− 6	3	1260	− 6.65	168
	# 29	1310	− 6	3	1260	− 6.65	378.5
	# 23	1310	− 6	3	1210	− 7.2	168
	# 30	1310	− 6	3	1210	− 7.2	672
	# 25.2	1310	− 6	3	1110	− 8.4	168
	# 32	1310	− 6	3	1110	− 8.4	672
FMQ	# 12	1310	− 7	6	zero time		
	# 13	1310	− 7	2			
	# 14	1310	− 7	3	1260	− 7.65	168
	# 27	1310	− 7	3	1260	− 7.65	672
	# 15.2	1310	− 7	3	1210	− 8.2	168
	# 28	1310	− 7	3	1210	− 8.2	672
	# 24.2	1310	− 7	3	1110	− 9.4	168
	# 33	1310	− 7	3	1110	− 9.4	672
FMQ -1	# 20	1310	− 8	3	1260	− 8.65	168
	# 31	1310	− 8	3	1260	− 8.65	671
	# 21	1310	− 8	3	1210	− 9.2	168.5
	# 34	1310	− 8	3	1210	− 9.2	672
	# 26	1310	− 8	3	1110	− 10.4	168
	# 35	1310	− 8	3	1110	− 10.4	672

The samples # 12 and # 13 are zero time experiments

### Analytical tools

#### X-ray micro-computed tomography (X-ray *µ*CT)

X-ray *µ*CT was performed at the Department of Geosciences and Environment at the University of Lausanne (UNIL) with a Bruker Skyscan© 1173 at an accelerating voltage of 40 kV, a current of 200 µA without a filter. Pixel size was varied between 5.5 and 6.0 *µm*, exposure time was set to 800 ms, the rotation step to 0.225° with accumulating 5–6 frames for each position. Image reconstruction was performed using the NRecon software and the Skyscan software package was used for 3D visualization. X-ray *µ*CT was employed to locate the diopside crystal seed in the quenched melt and to minimize the risk of destroying it during polishing. Additionally, as a diffusional anisotropy was expected to occur (Zhang et al. 2010), we were able to orient the sample prior to the polishing. The final goal was to cut the polished section such that it contained the long axis of the crystal, presumably the c-axis, which was best recognized and, therefore, the easiest recovered axis during the polishing (and another, undefined orientation perpendicular to the c-axis). The final polishing step was performed with a 1–0.25 *µm* polycrystalline diamond suspension. A detailed description of the polishing procedure is provided in the Electronic Appendix (incl. Fig. A1).

#### Electron backscatter diffraction (EBSD)

EBSD allowed the determination of the crystallographic orientation of the diopside crystal seed to define a suitable place to take the concentration profiles (along a certain axis) and to calculate the exact crystallographic direction of each profile within the crystal seed (detailed description in the Electronic Appendix; Fig. A2). All measurements were performed at the Scientific Center for Optical and Electron Microscopy (ScopeM) at ETH Zürich with an EBSD system (OIM 7 Pegasus with Hikari EBSD detector and Octane Super EDX detector; all by Ametek-EDAX) attached to a FEI Quanta 200F FEG-SEM with the following setting: tilt of the sample by 70°, acceleration voltage of 20 *kV* and low vacuum conditions (60 *Pa* as samples were non-carbon coated). The working distance varied between 15 and 20 *mm*. For EBSD pattern acquisition, the program OIM Data Collection was used; automated runs were performed at a speed of around 40–50 frames per second using camera pixel binning of 4 × 4. Subsequently, the program *OIM Analysis* was used to process the data. Cleaning of the data was performed based on (1) grain confidence index standardization and (2) neighbor orientation correlation with a grain tolerance angle of 5°. Further filtering was performed on a confidence index of 0.2 and grain size (variable for each sample) to extract the orientation map for the diopside crystal seed without surrounding matrix (Fig. A3).

#### Electron probe micro-analysis (EPMA)

EPMA measurements were conducted with a JEOL JXA-8200 electron microprobe equipped with five wavelength-dispersive spectrometers (WDS) at the Institute of Geochemistry and Petrology at ETH Zürich. The following standards were employed for major element analysis: fayalite for Fe, periclase for Mg, albite or acmite for Na, albite or anorthite for Al and wollastonite for Si and Ca. An acceleration voltage of 15 *kV*, a beam current of 20 *nA* with a focused beam for the crystal phases and a defocused beam with a spot size of 20 *µm* for the residual melt/interstitial eutectic mixture were employed. The counting time was 20 *s* on the peak and 10 *s* on the backgrounds. For Na the counting time was set to 10 *s* on the peak and 5 *s* on the backgrounds to minimize alkali loss. Measurements were performed to determine the composition of the vitrified starting material and of the phase assemblage in all experiments. To obtain statistically significant measurements, each phase was measured on ≥ 2 representative areas (diopside crystal seed) or crystals (anorthite, Ca-rich cpx or wollastonite) consisting of ≥ 8 measuring points.

#### Profiling with EDS using the FEG-SEM

**Fig. 2:**
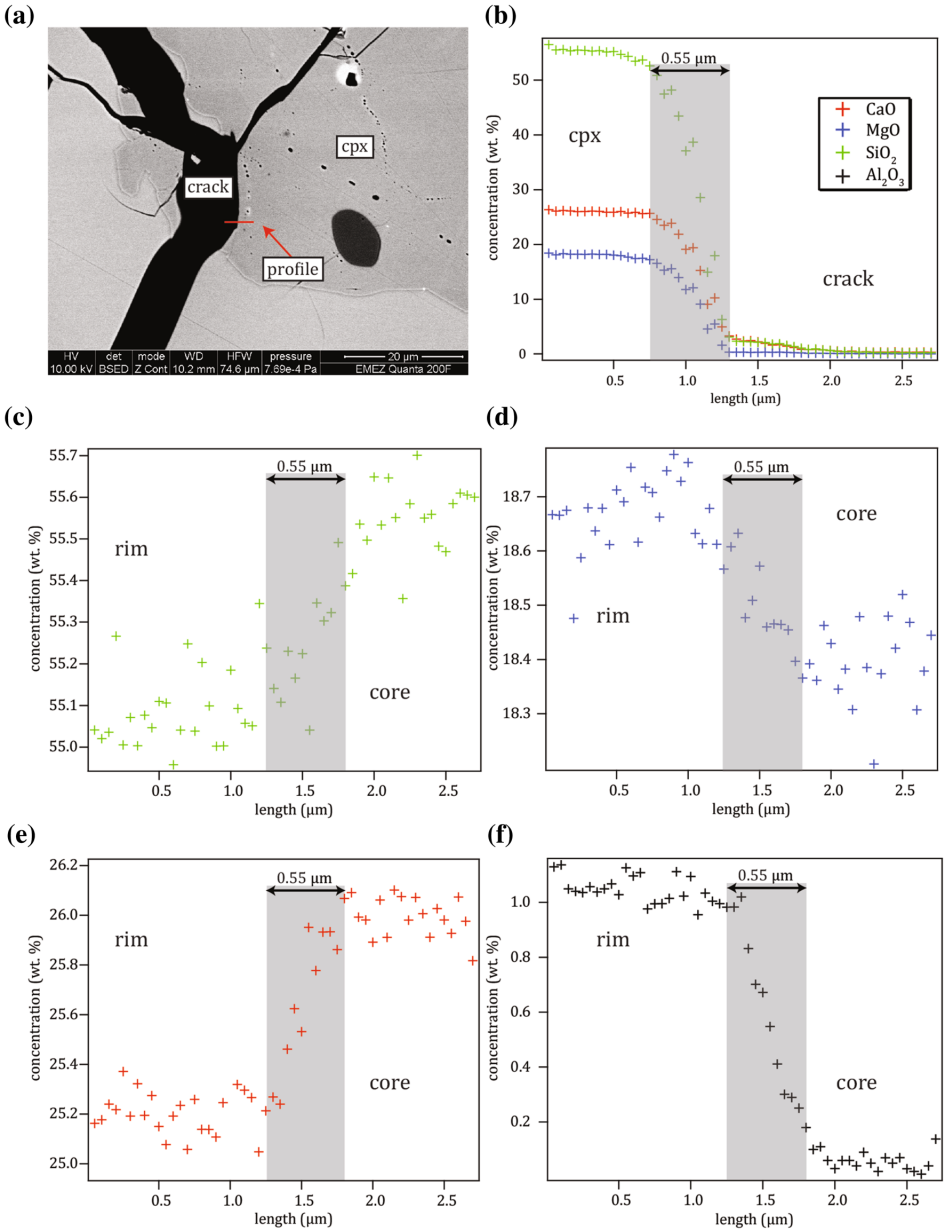
**a** BSE image of the location of the profile measured through the crack/crystal interface (profile shown in **b** which was used to parameterize the beam size in the Di/An system. **c**–**f** Zero-time experiment indicating similar profile length as in **b**

High resolution element diffusion profiles across the interface between the newly grown crystal rim and the diopside crystal seed were measured by quantitative EDS. The EDS system (TEAM 4.2 with Octane Super by Ametek-EDAX) was attached to a FEI Quanta 200F FEG-SEM at the ScopeM at ETH Zürich. The device was equipped with a Schottky type field emission source. The EDS “TEAM” software was used to define standards by re-analyzing the same areas/crystal that were previously measured by EPMA (no polishing after the EPMA measurement) and they were taken as ‘known’ concentrations: the diopside crystal seed was used for Si, Ca and Mg and anorthite crystals for Al (Fig. 1c, d). The reduced area mode was used to compensate for possible inhomogeneities in the crystal seed or the anorthite. Measuring time was 500 *s* for each area. By combining these two standards (cpx crystal seed and anorthite), one merged standard was created for each experiment. Both, standardization and diffusion profiles, were measured under identical operating conditions: 10 *kV* acceleration voltage, an EDS count rate of around 40,000 counts per second daily adjusted as no absolute beam current readings were available and an amp time of 1.92 *µs* resulting in a detector resolution of 135 *eV*. The profiles were always measured perpendicular to the rim/crystal interface (Fig. 1e, f). As the number of measurement points per line was limited to 20, 2–4 lines composed of 20 measurement points and a line length of 1–2 *µm* were placed consecutively as close as possible resulting in total profile lengths of 2–6* µm* (i.e., a lateral distance of 50–100 *nm* between neighboring points). Each point was measured for 30 *s*. Evaluation of the results of the zero-time experiments and of a measurement through the interface between a crack within the crystal seed (Sect. 2.4 and Fig. 2), allowed us to quantify the spatial resolution of the instrument that amounted to < 550 *nm* (full width at half maximum; Table A1).

### Data analysis

A common problem encountered with the analysis of diffusion profiles by electron micro-beam techniques is the apparent decrease in spatial resolution due to the finite size of the interaction volume. The measured profile results from the convolution of the true concentration profile with the beam interaction volume. In consequence, this leads to an overestimation of D (Ganguly et al. 1988). To reduce the influence of the finite interaction volume, we used a FEG-SEM operated at 10–12 *kV*. To deconvolve the measured profiles, the effective interaction volume, assuming a Gaussian distribution with radial symmetry about the beam axis, needs to be determined for each element. For this purpose, one profile was measured through the interface between the diopside crystal seed and an internal crack for the deconvolution of Si, Mg and Ca (Fig. 2). Ideally (with an infinitely small beam), such a profile (seed/crack) would show a step function shape instead of a curved one. For the deconvolution procedure, the measured curved profiles were fitted to a complementary error function (integral of Gaussian function) and its derivative with respect to the spatial variable yields the Gaussian beam profile which was used to deconvolve the diffusion profiles (fit coefficients in Table A1). For the cations Si, Ca and Mg, characteristic fits to the error function were established, whereas the Al concentration was too low for proper fitting and instead, we employed the effective interaction volume determined for Si.

**Fig. 3:**
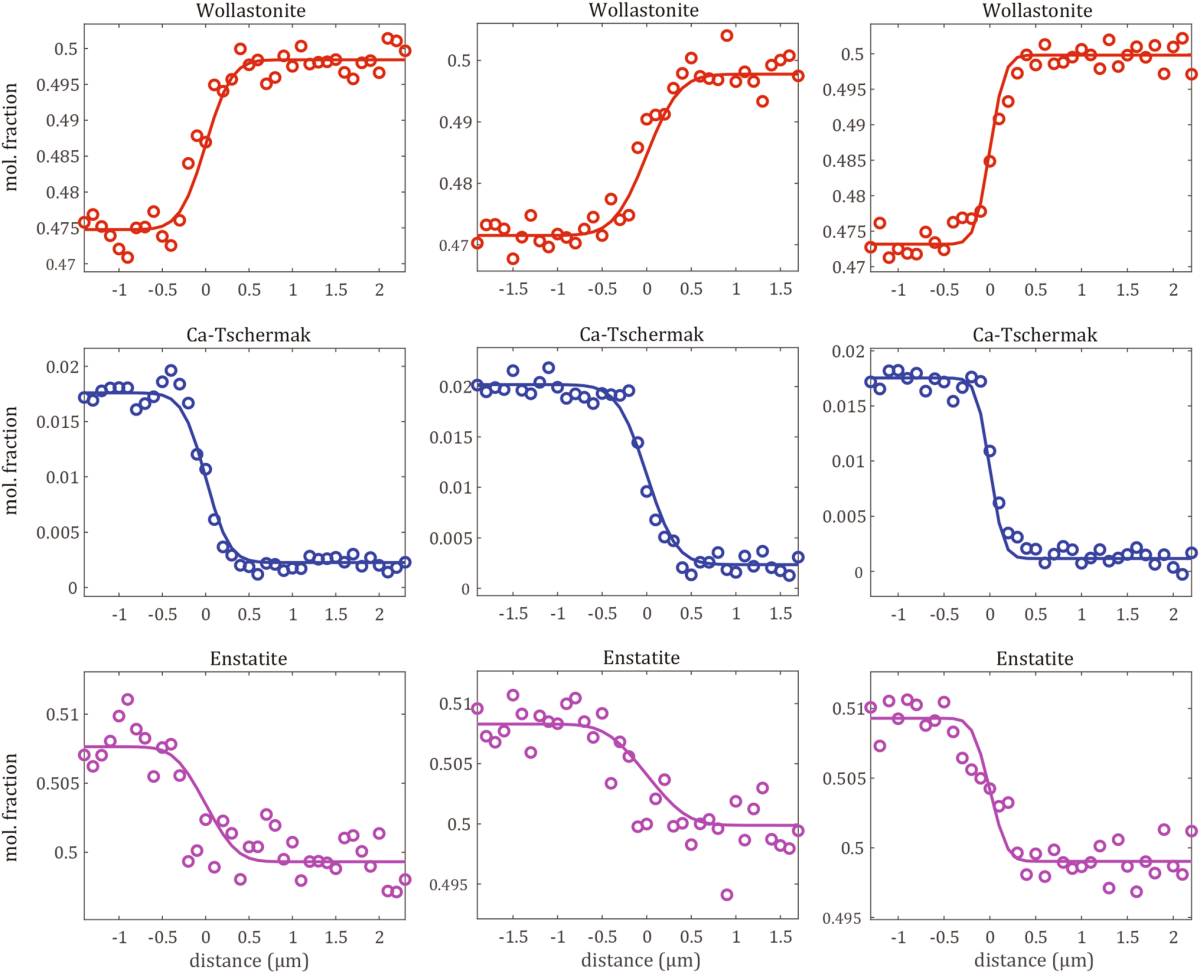
Three profiles from sample #29C (1260 °C, FMQ + 1, 378.5 h), #28C (1210 °C, FMQ, 672 h) and #35C (1110 °C, FMQ-1, 672 h). Circles represent the input data after deconvolution with the fitted solid lines; red representing Ca_2_Si_2_O_6_, blue CaAl_2_SiO_6_ and purple Mg_2_Si_2_O_6_

The end-member species of the ternary pyroxene(oid) system wollastonite, Ca-Tschermak´s pyroxene and enstatite were calculated based on the norm proposed by Wood and Banno (1973) using the element concentrations after deconvolution. By employing end-member species instead of elements, the requirement of preserving electron-neutrality during coupled diffusion is automatically satisfied. More importantly, with this approach the number of diffusing components was decreased from four (Al, Ca, Si and Mg) to three species with only two independent ones. Consequently, the diffusion matrix was reduced to a two-by-two matrix. The data analysis approach is based on the study of Trial and Spera (1994) employing a least-square minimization method to obtain the multicomponent diffusion matrix **D** (Trial and Spera 1994; Liang 2010; Claireaux et al. 2016). The model inputs are the deconvoluted species concentration profiles. The diffusion equation is expressed as follows:

1 $$\frac{{\partial w_{i} }}{\partial t} = \mathop \sum \limits_{j = 1}^{N} D_{ij} \frac{{\partial^{2} w_{j} }}{{\partial x^{2} }},$$

where *w*_*i*_ is the mass fraction (concentration) of chemical species *i*, *N* is the number of independent species (two in ternary pyroxene), *t* is the time (*s*), *D*_*ij*_ are the diffusion coefficients (*m*^*2*^ *s*^−*1*^) and *x* the spatial coordinate ($m$). The diffusion matrix ***D*** can be diagonalized using:

2 $$D = P\varLambda P^{ - 1} ,$$

where the columns of ***P*** are the eigenvector matrix of ***D***, λ_i_ are the eigenvalues of ***D*** and *Ʌ* = diag (λ_i_) (Trial and Spera 1994). The eigenvector of the larger eigenvalue indicates which chemical species exchange is dominant and the eigenvalue indicates its rate (Claireaux et al. 2016). Eigenvalues must be positive as they are considered as diffusion coefficients and the diagonalization is a transformation of the coordinate system where the axes become independent component concentrations (Trial and Spera 1994). The solution to Eq. 1 is the following:

3 $$w_{i} = w_{i}^{\text{bulk}} + \mathop \sum \limits_{j = 1}^{N} \mathop \sum \limits_{k = 1}^{N} P_{ij} f_{i} P_{jk}^{ - 1} \varDelta w_{k} ,$$

where *w*_*i*_ are the modeled concentrations and *∆w*_*k*_ are the initial concentration differences across the couple. For short time, the couple is defined as an infinite medium with the following definitions:

4 $$w_{i}^{bulk} = \frac{{w_{i}^{\left( 1 \right)} + w_{i}^{\left( 2 \right)} }}{2},$$

5 $$f_{i} = - \frac{1}{2}{\text{erf}}\left( {\frac{x}{{2\sqrt {\lambda_{i} t} }}} \right),$$

where $w_{i}^{\text{bulk}}$ is the bulk composition of the couple and ${\text{erf}}$ is the error function. Finally, the Chi square criterion by Press et al. (1986) was applied that minimizes the differences between the modeled and the measured concentrations:

6 $$\chi^{2} = \mathop \sum \limits_{j = 1}^{M} \mathop \sum \limits_{i = 1}^{N + 1} \left( {\frac{{c_{i} \left( {x_{j} ,t_{j} } \right) - w_{i} \left( {\varvec{a};x_{j} ,t_{j} } \right)}}{{\sigma_{i, j} }}} \right)^{2} ,$$

where *w*_*i*_*(****a****;x*_*j*_*,t*_*j*_*)* are the concentrations predicted by the model, ***a*** contains the unknown parameters (eigenvalues and eigenvectors of ***D***), *c*_*i*_*(x*_*j*_*,t*_*j*_*)* correspond to the measured concentrations, *M* is the number of FEG-SEM measuring points and *σ*_*i,j*_ is the uncertainty of the concentration (subsequently referred to as sigma). Further details of the model are presented in the Electronic Appendix. To demonstrate the reliability of our model approach, three concentration profiles with their respective fits are presented in Fig. 3. Additionally, the Boltzmann–Matano plane is required (the location in the diffusion profile where the two integrals right and left from this interface are summing up to 0 accordingly to their mass balance) to determine the initial profiles using the following equation:

7 $$\mathop \smallint \limits_{{r_{ \hbox{min} } }}^{{r_{ \hbox{max} } }} \left[ {c_{i} \left( {r, t} \right) - c_{i} \left( {r,0} \right)} \right]{\text{d}}x = 0,$$

where *r*_max_ and *r*_min_ are the coordinates at the beginning and the end of the profile and *x* is the coordinate along the diffusion profile (Vielzeuf and Saúl 2011). We used a step function as starting point of the model where the starting values were calculated by averaging the two plateaus (rim and core) for each species. Sigma on each plateau was calculated based on the standard deviation of the absolute difference between the (calculated) plateau and the measurements.

The uncertainties of the eigenvalues and eigenvectors were determined by a Monte-Carlo approach (Anderson 1976; Trial and Spera 1994; Liang et al. 1996; Freda et al. 2005). The previously determined sigma of each species was used as a Gaussian noise added on the measured profiles as a representation of the analytical errors. The least-square minimization was repeated in the Monte-Carlo algorithm for ≤ 500 times. A MATLAB script was used to retrieve the diffusion matrix of each experiment. However, the random input data were based on the plateaus which were in some experiments relatively noisy resulting in physically incorrect fits during the Monte-Carlo simulation. These outliers were removed from the final histograms.

The modeling strategy was the following: first, we tested several P value combinations for all experiments with the aim a) to obtain good quality fits for each single profile and b) to minimize the coupling effect. Subsequently, we re-run the model for all experiments implementing the pre-determined global P matrix to calculate the eigenvalues. This procedure revealed that *λ1* is approximately a quarter order of magnitude larger than *λ2*; therefore, we re-run the model again with a fixed ratio between *λ1* and *λ2* of 0.25 to determine the optimal *λ1* values.

## Results

### Starting material and phase assemblage

The starting material has a uniform composition of 69 wt. % diopside and 29 wt. % anorthite (Table 1). The starting material has a slight Fe_2_O_3_ (0.25 wt. %) contamination which was most likely introduced by impure, commercially available CaSiO_3_ (as later confirmed by XRF-analyses of the powder).

**Fig. 4:**
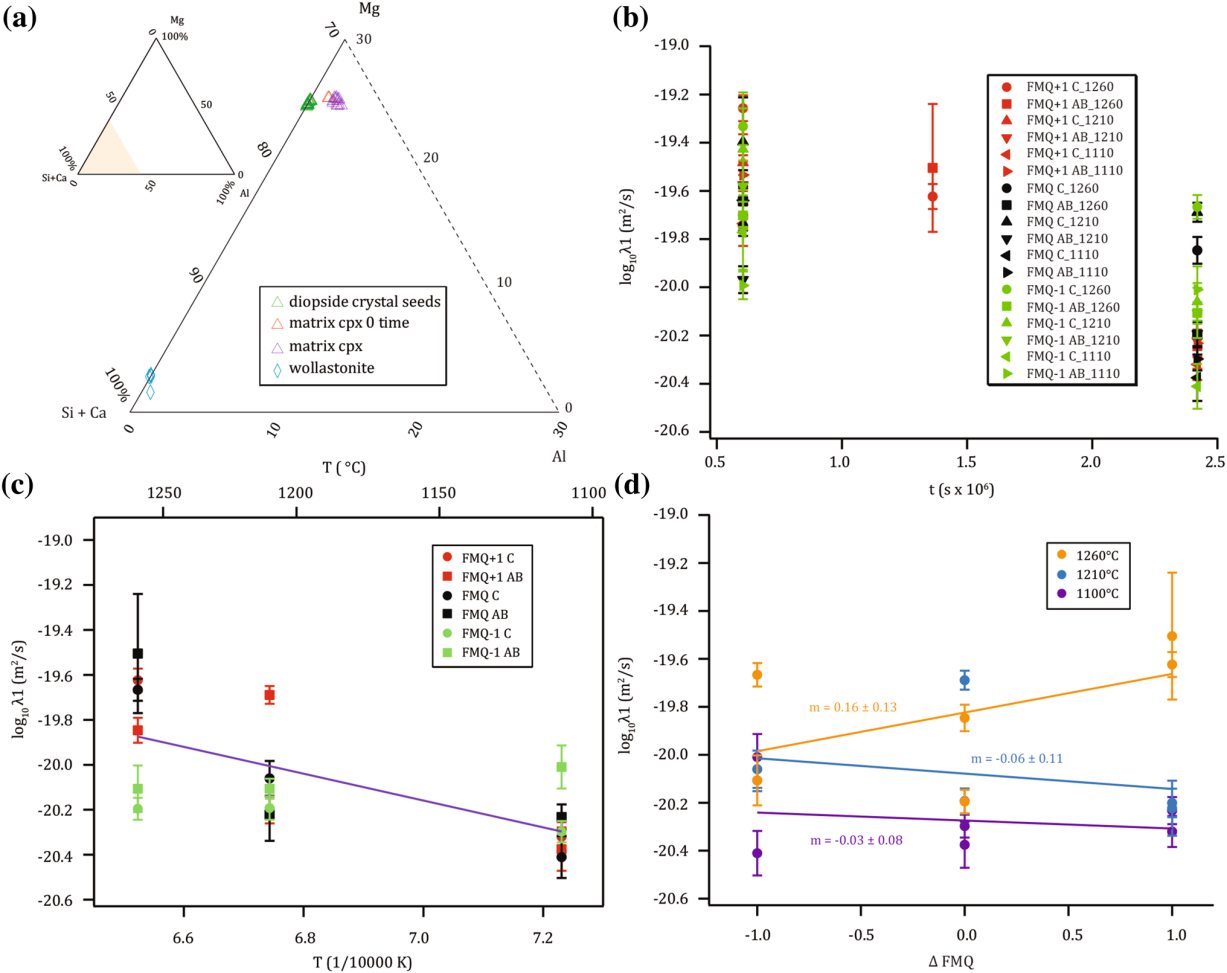
**a** Ternary pyroxene diagram displaying averaged measurements of Table A2 illustrating the compositions of the diopside seeds, the Ca-rich matrix cpx and the sporadically occurring wollastonites. **b** Eigenvalues (*λ*1) of all experiments as a function of run duration indicating time-dependent behavior. **c** Arrhenius diagram of *λ1* based on the longest-duration experiments. The fitted line is the Arrhenius equation corresponding to Eq. 8. **d** Eigenvalue (*λ*1) of long-duration experiments as a function of *fO*_*2 *_^(ΔFMQ)^

The phase assemblage of the experiments consisted of the added diopside crystal seed (sensu stricto), anorthite plagioclase, Ca-rich cpx (first and second generation), residual melt (at *T* of 1310 °C) and fine-grained “interstitial” quench mineral phases formed upon eutectic crystallization of the interstitial liquid present at 1310 °C during *T* decrease below the solidus *T* when setting the run conditions for the diffusion annealing experiment (Fig. 4a). Occasionally, some rare wollastonite was observed in the fine-grained matrix (Fig. 1c). Representative analyses of each phase are provided in Table 3 and all averaged EPMA data are given in Table A2 (except for the fine-grained eutectic mixtures). The averaged composition of all diopside crystal seeds confirms its near perfect stoichiometry (Na_0.002_Ca_1.003_Mg_0.991_Fe_0.004_Al_0.002_Si_1.999_O_6_) basically devoid of Na, Fe or Al. In contrast, anorthite crystals vary in composition, size and quantity between each experiment depending on *T*, covering a fairly broad compositional range of Na_0.000–0.064_Ca_0.949–1.009_Al_1.628–1.939_Fe_0.000–0.008_Mg_0.028–0.124_Si_2.024–2.257_O_8_ (Na component was introduced by contamination from the furnace insolation tube). Wollastonite was sporadically observed with the composition Na_0.000–0.001_Ca_0.932–0.950_Al_0.001–0.013_Mg_0.033–0.057_Si_0.991–1.002_O_3_. Residual melt compositions are not discussed here but provided in Table A2.

**Table 3:** EMPA measurements of representative mineral phases in *wt.*  *%* and cations in *p.f.u*

wt. %	Di. crystal		# 13		# 32		# 26		# 28	
	seeds		matrix cpx		matrix cpx		anorthite		wollastonite	
	avg. (729)	std.	avg. (21)	std.	avg. (24)	std.	avg. (30)	std.	avg. (9)	std.
SiO_2_	55.37	0.22	54.42	0.20	53.75	0.70	45.11	0.37	51.59	0.15
Al_2_O_3_	0.06	0.10	1.21	0.09	3.06	0.99	34.13	0.71	0.11	0.03
FeO	0.10	0.07	0.08	0.01	0.13	0.04	0.05	0.02	0.02	0.02
MgO	18.44	0.19	18.70	0.08	18.02	0.59	0.53	0.06	2.01	0.03
CaO	25.94	0.19	24.73	0.10	24.95	0.24	19.96	0.51	46.16	0.13
Na_2_O	0.02	0.02	0.01	0.01	0.01	0.01	0.22	0.04	0.00	0.00
Total	99.92	0.35	99.40	0.18	99.91	0.54	100.00	0.58	99.89	0.25
cation										
Si	2.00		1.97		1.94		2.09		0.99	
Al	0.00		0.05		0.13		1.86		0.00	
Fe	0.00		0.00		0.00		0.00		0.00	
Mg	0.99		1.01		0.97		0.04		0.06	
Ca	1.00		0.96		0.96		0.99		0.95	
Na	0.00		0.00		0.00		0.02		0.00	
Total	4.00		4.00		4.00		5.00		2.00	

Numbers in brackets denote number of measurements

*Di.* indicates diopside, *std.* one-sigma standard deviation, *avg.* average

Two generations of rim and matrix cpx are present. The ***first*-*generation cpx*** occurs (1) as individual, unzoned crystals in experiments which were not exposed to a *T* reduction step (e.g., zero-time experiments) and (2) as cores in the experiments that were exposed to the *T* reduction step. They form the first-generation rims that mantle the diopside crystal seed. They have the same composition as the first-generation matrix cpx and constitute the Al-rich part of the diffusion couples. The ***second*-*generation matrix cpx*** and rims were only present in those experiments where *T* was reduced. They occur either (1) as individual crystals, (2) as rims around the first-generation cpx (now being the cores) and (3) as a second overgrowth rim on the first overgrowth mantle around the diopside crystal seed. The diffusion couples evaluated in this study were not affected by the second-generation rim growth as the first rim (mantle) constituting parts of the diffusion couple that was always produced at a constant *T* (1310 °C) with a constant composition and, therefore, “sealed” off the crystal seed from later grown rims. The two compositions were indistinguishable in the BSE images but were clearly identified from the EDS profiling (Fig. A4). The composition of the first-generation cpx is precisely known from the “zero” time experiment (# 13; Fig. 2c – f) providing the composition Na_0.000_Ca_0.961_Mg_1.011_Fe_0.002_Al_0.05_Si_1.973_O_6_ characterized by an increase in Al and Mg contents and decreasing Si and Ca contents in comparison with the diopside crystal seeds. The abundance of the first-generation cpx in the “zero” time experiment was relatively low (< 10 vol. % and without coexisting anorthite) dictated by the bulk composition in the binary diopside–anorthite system (Osborn 1942). The second-generation cpx formed during the diffusion anneal at lower *T* and they reveal a relatively broad compositional range of Na_0.000–0.001_Ca_0.946–0.973_Mg_0.967–1.012_Fe_0.001–0.005_Al_0.067–0.130_Si_1.936–1.973_O_6_. The difference to the first-generation cpx is attributed to cpx and anorthite growth due to the *T* reduction after 3 h at a *T* of < 1270 °C, i.e., below the eutectic temperature where the remaining liquid crystallized to an intimate mixture of the two phases (referred to as “interstitial”). Most likely, there are also first-generation cpx in the eutectic mixture that were overgrown by second generation ones but this was not confirmed by EPMA (quantitatively or visibly).

### EBSD and diffusion matrix

219 diffusion profiles (varying measuring step size of 50–100 *nm*) from 18 experiments were measured and 82 were considered for the calculation of the eigenvalues and eigenvectors for the coupled diffusion of the species: wollastonite, Ca-Tschermak and enstatite. Enstatite was chosen as the dependent species. As pointed out by Claireaux et al. (2016), the eigenvectors indicate the chemical species exchange and the eigenvalues indicates the corresponding exchange rate.

Our data reveal a slight time-dependency for *λ*1 with an averaged difference of 0.44 log units between shortest and longest run times (Table 4; Fig. 4b). Within the errors, several experiments overlap, however, this time-dependent behavior is consistent for all experiments (with one exception). To minimize the risk of calculating erroneous results, we excluded the eigenvalues of the short-duration experiments for the calculation of the Arrhenius equations (Fig. 4c). It can be expected that the time-dependency decreases with longer run times in an exponential way. However, we are not entirely sure if our long-duration experiments are indeed completely devoid of any time-dependent effects inferring that our eigenvalues can only be considered as upper values. From our point of view, this slight time-dependence is due to fact that the measured profiles are extremely short and, thus, the applied deconvolution did not completely erase the overlap of the interaction volumes of the beam (Fig. A5 presents profiles before and after deconvolution). This assumption is supported by the observation that the difference between long- and short-duration experiments is decreasing with increasing *T* which is related to the broader profiles generated in the high *T* experiments rendering them less vulnerable for the intrinsic resolution problem.

**Table 4:** Summary of EBSD measurements indicating the averaged angular deviation of measured profiles relative to the closest crystal axes and results of matrix inversion

fO_2_	run &	#	offset		coefficient of the eigenvectors (normalized)								log_10_ eigenvalue (*m*^*2*^*/s*)			
	axis	pr.	(°)	std.	P11	std.	P21	std.	P22	std.	P12	std.	λ1	std.	λ2	std.
FMQ +1	# 22.2C	2	12.6	0.0	1.00	0.00	− 0.38	0.00	1.00	0.00	− 0.65	− 0.01	− 19.26	0.05	− 19.51	0.05
	# 29C	7	11.0	4.2	1.00	0.01	− 0.42	− 0.02	1.00	0.04	− 0.87	− 0.04	− 19.62	0.05	− 19.87	0.05
	# 22.2B	2	24.1	0.0	1.00	0.00	− 0.37	0.00	1.00	0.00	− 0.64	0.00	− 19.64	0.11	− 19.89	0.11
	# 29B	1	24.9	0.0	1.00	0.01	0.25	0.02	1.00	0.00	− 0.62	0.00	− 19.50	0.27	− 19.75	0.27
	# 23C	3	10.8	5.4	1.00	0.04	− 0.27	− 0.20	1.00	0.21	− 0.74	− 0.46	− 19.48	0.12	− 19.73	0.12
	# 30C	1	19.9	0.0	1.00	0.03	− 0.35	− 0.07	1.00	0.32	0.00	− 0.65	− 20.20	0.06	− 20.45	0.06
	# 23A	3	17.1	1.4	1.00	0.04	− 0.39	− 0.08	1.00	0.13	− 0.74	− 0.15	− 19.57	0.12	− 19.82	0.12
	# 30B	1	27.5	0.0	1.00	0.07	− 0.49	− 0.11	1.00	0.28	− 0.39	− 0.91	− 20.22	0.11	− 20.47	0.11
	# 25.2C	3	17.2	3.3	1.00	0.21	− 0.44	− 0.18	1.00	0.83	− 0.37	− 0.94	− 19.74	0.09	− 19.99	0.09
	# 32C	4	11.5	2.8	1.00	0.12	-0.14	-0.45	1.00	0.14	−0.99	− 0.14	− 20.32	0.06	−20.57	0.06
	# 25.2B	2	29.4	5.8	1.00	0.06	− 0.41	− 0.10	1.00	0.12	− 0.78	− 0.12	− 19.53	0.06	− 19.78	0.06
	# 32A	3	14.2	2.5	1.00	0.08	− 0.20	− 0.25	1.00	0.10	− 0.95	− 0.10	− 20.23	0.06	− 20.48	0.06
FMQ	# 14C	3	12.2	0.9	1.00	0.03	− 0.52	− 0.04	1.00	0.11	− 0.77	− 0.19	− 19.74	0.04	− 19.99	0.04
	# 27C	1	5.9	0.0	1.00	0.02	− 0.39	− 0.04	1.00	0.17	− 0.60	− 0.42	− 19.85	0.06	− 20.10	0.06
	# 14B	1	14.1	0.0	1.00	0.00	− 0.35	0.00	1.00	0.00	− 0.63	0.00	− 19.64	0.05	− 19.89	0.05
	# 27B	2	46.1	1.9	1.00	0.02	− 0.74	− 0.03	1.00	0.08	− 1.19	−0.09	− 20.20	0.05	− 20.45	0.05
	# 15.2C	2	3.6	2.3	1.00	0.03	− 0.41	− 0.06	1.00	0.12	− 0.62	− 0.27	− 19.40	0.18	− 19.65	0.18
	# 28C	2	23.7	0.4	1.00	0.02	− 0.56	− 0.02	1.00	0.30	0.08	0.63	− 19.69	0.04	− 19.94	0.04
	# 15.2A	1	20.3	0.0	1.00	0.05	− 0.47	− 0.10	1.00	0.07	− 0.73	− 0.08	− 19.97	0.06	− 20.22	0.06
	# 28AB	3	16.2	1.9	1.00	0.04	− 0.43	− 0.13	1.00	0.18	− 0.52	− 0.34	− 20.19	0.05	− 20.44	0.05
	# 24.2C	2	24.7	0.0	1.00	0.00	− 0.33	0.00	1.00	0.01	− 0.65	− 0.01	− 19.64	0.12	− 19.89	0.12
	# 33C	6	8.6	2.3	1.00	0.02	− 0.32	− 0.05	1.00	0.12	− 0.85	− 0.30	− 20.38	0.10	− 20.63	0.10
	# 24.2A	1	23.2	0.0	1.00	0.00	− 0.34	0.00	1.00	0.00	− 0.63	0.00	− 19.64	0.07	− 19.89	0.07
	# 33A	2	21.8	7.0	1.00	0.05	-0.49	-0.10	1.00	0.08	− 0.92	− 0.10	− 20.30	0.05	− 20.55	0.05
FMQ -1	# 20C	1	19.5	0.0	1.00	0.00	-0.35	0.00	1.00	0.01	− 0.64	− 0.01	− 19.33	0.14	− 19.58	0.14
	# 31C	2	11.8	0.3	1.00	0.02	− 0.55	− 0.03	1.00	0.07	− 0.73	− 0.09	− 19.67	0.05	− 19.92	0.05
	# 20A	1	40.7	0.0	1.00	0.10	− 0.45	− 0.13	1.00	0.18	− 0.75	− 0.19	− 19.70	0.06	− 19.95	0.06
	# 31B	1	34.2	0.0	1.00	0.02	− 0.65	− 0.03	1.00	0.08	− 1.41	− 0.16	− 20.11	0.10	− 20.36	0.10
	# 21C	3	13.9	0.1	1.00	0.01	− 0.38	− 0.02	1.00	0.04	− 1.16	− 0.04	− 19.43	0.17	− 19.68	0.17
	# 34C	1	16.9	0.0	1.00	0.03	− 0.40	− 0.06	1.00	0.04	− 0.68	− 0.04	− 20.06	0.08	− 20.31	0.08
	# 21A	1	38.9	0.0	1.00	0.01	− 0.37	− 0.02	1.00	0.01	− 0.64	− 0.01	− 19.58	0.17	− 19.83	0.17
	# 34AB	4	29.0	11.2	1.00	0.14	0.09	0.41	1.00	0.12	− 0.95	− 0.12	− 20.11	0.04	− 20.36	0.04
	# 26C	2	8.2	0.0	1.00	0.02	− 0.35	− 0.05	1.00	0.18	− 0.57	− 0.43	− 19.77	0.15	− 20.02	0.15
	# 35C	5	8.6	0.6	1.00	0.03	− 0.54	− 0.04	1.00	0.06	− 1.45	− 0.06	− 20.41	0.09	− 20.66	0.09
	# 26A	1	41.9	0.0	1.00	0.00	− 0.35	0.00	1.00	0.00	− 0.62	0.00	− 19.99	0.06	− 20.24	0.06
	# 35A	2	25.6	0.3	1.00	0.02	− 0.56	− 0.03	1.00	0.42	0.39	0.68	− 20.01	0.10	− 20.26	0.10

For presentation purposes, the eigenvector coefficients were normalized to P11 and P22. The λ1 and λ2 values were determined by re-modelling all the experiments with fixed P11, P12, P21 and P22 values of 1, − 0.38, − 0.67 and 1. λ1 and λ2 are the first and second eigenvalue; *A, B and C* indicate the crystallographic axes (AB means that both axes were present due to the occurrence of multi-grain crystals). *# pr.* refers to number of profiles, *offset* indicates the averaged angular deviation for the number of profiles with the one-sigma standard deviation (std.); the standard deviation from the eigenvalues and eigenvectors were derived by Monte-Carlo simulations. P11 and P21 normalized to P11 are the eigenvectors of v1 and P12 and P22 normalized to P22 are the eigenvectors of v2

For the purpose of detecting a possible diffusion anisotropy along different crystallographic axis, 50 profiles were measured along the c-axis and 32 sub-perpendicular to the c-axis (a^*^/b-axis) (Table 4; long-duration experiments in Fig. 4c). For the calculation of the eigenvalues no distinction was made between profiles measured along the a- and b-axis, which are practically treated as one axis. The EBSD measurements indicate that the deviation of the diffusion profile from the c-axis was in most cases relatively small, thus, confirming that the pre-orientation step with the X-ray *µ*CT worked well (Electronic Appendix). In both systems, most of the diopside crystal seeds are multi-grain aggregates composed of several (sub)-grains (Fig. A3). The averaged deviation between the profile lines and the c-axis is 12.2˚ ± 5.5˚. The second axis in the section plane varies between the a- and b-axis without any preference as the crystal shape is not very distinctive between these two axes. Thus, it was impossible to predict a priori which axis would be sectioned by polishing. For these axes, the deviation to the profiles amounted to 25.6˚ ± 10.4˚, which is considerably larger compared to the c-axis. The effect of small orientation deviation does not significantly influence the computed eigenvalues and eigenvectors. Therefore, no further re-mounting and re-orienting were performed to prevent further thinning-out of the crystals with the risk of losing the entire experiment (and an improvement of the orientation precision was not guaranteed). The *λ1* values parallel to the c-axis and perpendicular to the c-axis overlap, implying that diffusion along the c-axis occurs at the same rate as perpendicular to it suggesting that the coupled diffusion is isotropic within the resolution of this study (Fig. 4c).

Three different *fO*_*2*_ conditions were imposed (FMQ ± 1) to explore the effect of *fO*_*2*_ on diffusion. By adjusting the H_2_/CO_2_ ratio according to the experimental *T*, rim growth and diffusion anneal were performed under identical *fO*_*2*_ conditions relative to a solid buffers which is strongly advised (e.g., Cherniak and Dimanov (2010)). An exponential *fO*_*2*_ dependence with a power exponent m expressed as $fO_{2}^{m}$ in the range of 0.021 ± 0.11 (averaging the values from Fig. 4d) was determined for *λ1*. However, as this *fO*_*2*_ dependence is very inconsistent for different *T* for *λ1* (Fig. 4d), we calculated the Arrhenius equations without an *fO*_*2*_ term and based it only on results from the long-duration experiments. The Arrhenius equations for *λ1* and *λ2* are derived from a global fit through all long-duration experiments (Fig. 4c) resulting in the following equations (*λ2* is based on a fixed ration of 0.25 so that the *λ2* values plot at the same locations in the Arrhenius diagram just shifted 0.25 log units along the y-axis and are, therefore, not shown):

8 $$\lambda 1 = 10^{ - 15.98 \pm 1.17} \times \exp \left[ {\frac{{ - 114.4 \pm 32.8 {\text{kJ/mol}}}}{RT}} \right],$$

9 $$\lambda 2 = 10^{ - 16.23 \pm 1.17} \times \exp \left[ {\frac{{ - 114.4 \pm 32.8 {\text{kJ/mol}}}}{RT}} \right],$$

where *λ*1 and *λ*2 are the eigenvalues in m^2^ s^−1^, *R* is the gas constant (8.3144 J mol^−1^ K^−1^) and *T* the absolute temperature in *K*.

## Discussion

### Exchange mechanism

**Fig. 5:**
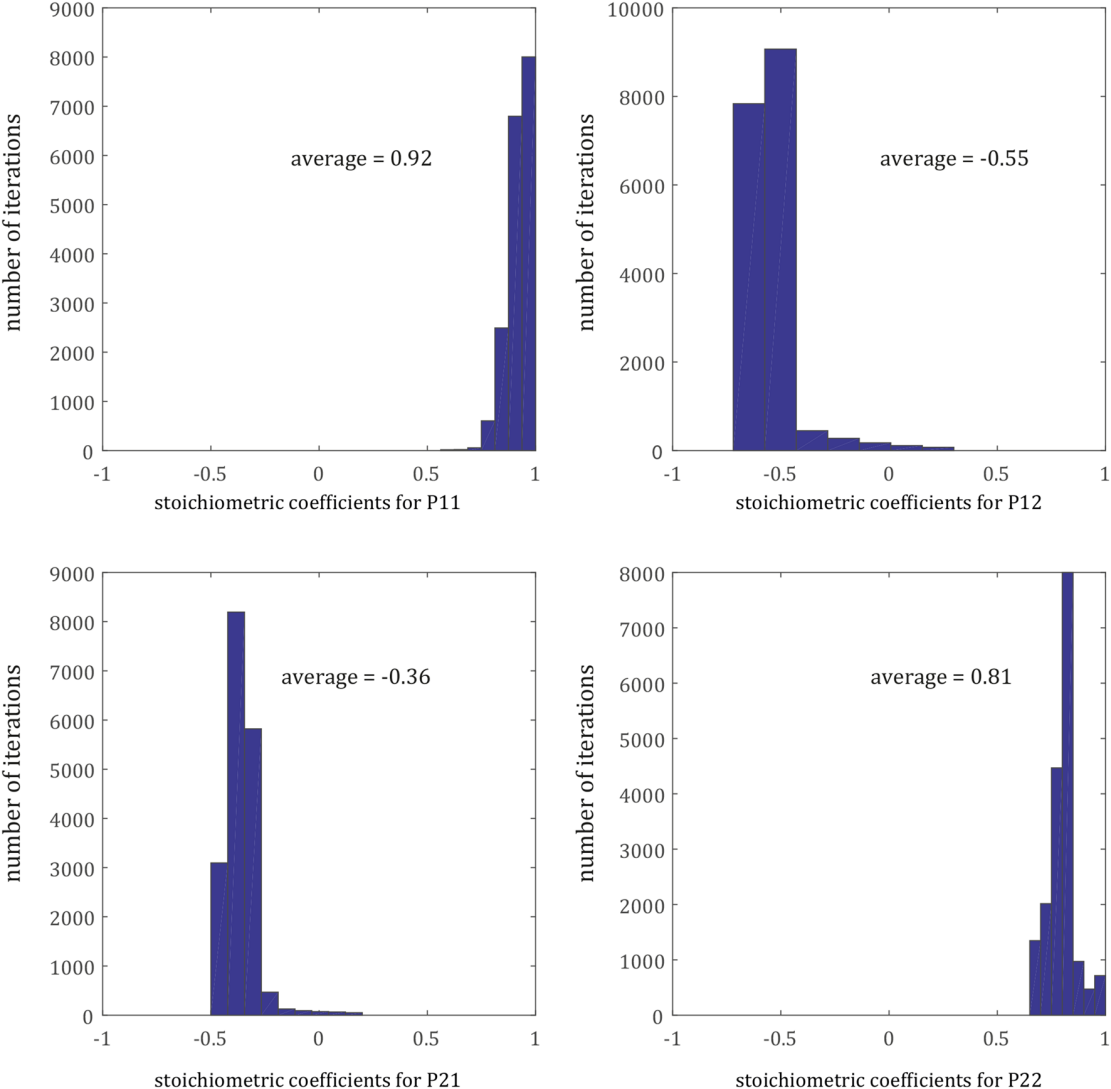
Histograms of the results for the single (non-normalized) stoichiometric coefficients of the exchange reaction based on all experiments (Monte-Carlo simulations are grounded on 500 iterations per experiment)

The dominant eigenvalue *λ1* is about one quarter order of magnitude larger than *λ2* and the determined eigenvectors indicate similar exchange mechanisms for all the different experiments regardless of run duration, *fO*_2_ and *T* (Table 4; Fig. 5). For presentation purposes (Table 5 and Fig. A6), the major diagonal eigenvector coefficients (P11 and P22) were normalized to one. By following the work of Chakraborty et al. (1995a) the exchange reactions are the following:

10 $$v1 \left( {\text{Di/An}} \right):1.00 {\text{Ca}}_{ 2} {\text{Si}}_{ 2} {\text{O}}_{ 6} { + 0} . 6 7 {\text{CaAl}}_{ 2} {\text{SiO}}_{ 6} \rightleftharpoons 1. 6 7 {\text{Mg}}_{ 2} {\text{Si}}_{ 2} {\text{O}}_{ 6} ,$$

11 $$v2 \left( {\text{Di/An}} \right):1.00 {\text{CaAl}}_{ 2} {\text{SiO}}_{6} + 0.38 {\text{Ca}}_{ 2} {\text{Si}}_{ 2} {\text{O}}_{6} \rightleftharpoons 1.38 {\text{Mg}}_{ 2} {\text{Si}}_{ 2} {\text{O}}_{6} .$$

**Table 5:** Summary of the diffusion parameters

parameter	70 Di–30 An			
	*λ* 1		*λ* 2	
	avg.	std.	avg.	std.
log_10_ D_O_ (m^2^/s)	− 15.98	1.17	− 16.23	1.84
Q (kJ/mol)	114.40	32.80	114.40	32.80
P11/P11	1.00	0.05		
P21/P11	− 0.38	0.03		
P22/P22			1.00	0.18
P12/P22			− 0.67	0.14

*λ1* and *λ2* refer to the first and second eigenvalue. *Q* is the activation energy and Do the pre-exponential factor. *P11* and *P21* are the coefficients of the first and P12 and P22 of the second eigenvector. *std.* is the one-sigma standard deviation

**Fig. 6:**
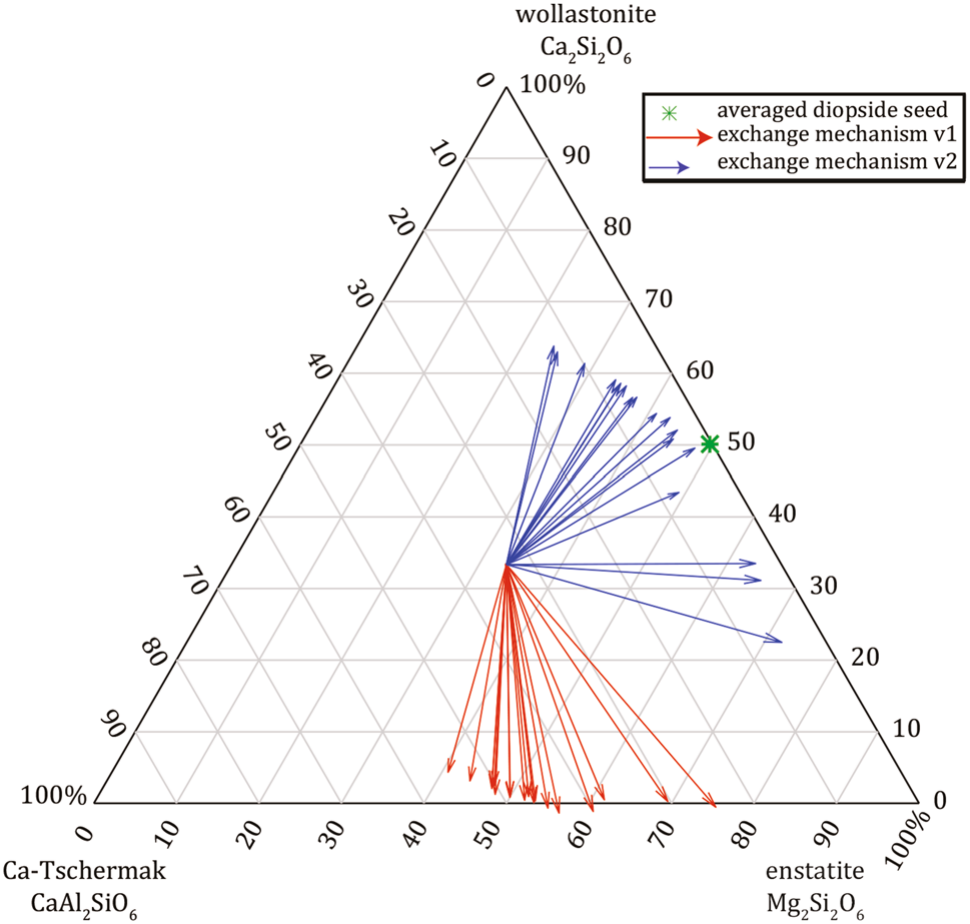
Ternary diagram (wollastonite—Ca-Tschermak—enstatite) illustrating the direction of exchange of the chemical species from an imaginary starting point (1/3, 1/3, 1/3); red vectors are *v1* and blue vectors *v2*. The length of the vectors is proportional to the rate of exchange derived from *λ1* and *λ2 *with a 1:4 ratio. For reference, the composition of the diopside crystal seeds is indicated with a green star

The uncertainties associated with the stoichiometric coefficients are provided in Table 5. The first exchange (Eq. 10) is very reproducible also with respect to the uncertainty, whereas *v2* shows a larger scatter (Eq. 11). Figure 6 illustrates the computed eigenvectors of the Di/An system in a ternary diagram in terms of end-member chemical species. Regardless of the measured four elements in the cpx solid solutions, only two parameters (*λ1* and *λ2*) are sufficient to characterize the coupled diffusion processes as *v1* and *v2* are constant. This is a significant advantage of performing the eigenvalue decomposition as it reduces the complexity and potentially provides critical insights into the nature of coupled diffusion in cpx.

The detailed exchange mechanisms are rather uniform for the investigated system which is linked to the specific setup of the diffusion couples. As shown by Trial and Spera (1994), different (perpendicular) compositional ranges are necessary to fully recover the entire D matrix inferring that additional experiments in other directions with contrasting diffusion couples would be required. However, for solid-state diffusion in minerals a considerably more elaborate experimental setup would be necessary compared to melt-based diffusion experiments (e.g., Claireaux et al. (2016)) because the compositions of the diopside crystal seeds predefine the entire diffusion couple. It is impossible to grow a rim with a “negative concentration” (e.g., the Ca-Tschermak´s species).

We are confident that the determined D matrix can be employed for the calculation of the duration that elapsed since a change in the physio-chemical environment in the (igneous) system occurred that led to an overgrowth rim in crystals with similar compositions as in our experiments (further details in Sect. 4.4).

### Effect of crystallographic axes and *fO*_*2*_

Considering the crystallographic structure of high Ca–cpx, a diffusional anisotropy is anticipated with diffusion along the c-axis being fastest. The diopside structure can conceptually be envisaged as a so-called I-beam structure where one I-beam unit consists of two tetrahedral units (2 tetrahedra) pointing inwards and cross-links to the octahedral cations on the M1 and M2 sites. The I-beam units extend along the c-axis forming chains of SiO_4_ tetrahedra (Thompson 1970; Papike et al. 1973), i.e. the structure is highly anisotropic. However, to date an unequivocal experimental verification is lacking and also the present results do not clarify the situation as they reveal no significant diffusional anisotropy. The only reliable data reporting anisotropy in high Ca–cpx is the study by Zhang et al. (2010) who demonstrated a diffusional anisotropy for ^44^Ca and ^26^Mg self-diffusion between 950 °C and 1150 °C with the relationship b < a^*^ < c (^26^Mg diffusion data had to be corrected to follow the compensation law). They observed a difference of 0.6 log units between the b- and c-axis at 1050 °C that nearly completely vanished at 1150 °C which is close to the lower *T* limit of our study. Other studies identified diffusional isotropy such as Dimanov et al. (1996) and Dimanov and Jaoul (1998) for ^44^Ca self-diffusion. Pacaud (1999) and Gasc et al. (2006) investigated ^26^Mg self-diffusion in natural and synthetic diopsides and Pacaud (1999) reported a D of 10^−18^ *m*^2^ *s*^−1^ at 1200 °C without any anisotropy. Gasc et al. (2006) reported a diffusion coefficient of 10^−19^* m*^2^ *s*^−1^ for synthetic and natural samples and Cherniak and Dimanov (2010) mentioned that isotropic diffusion behavior was observed in the synthetic sample of Gasc et al. (2006) but a slight diffusion anisotropy in the natural sample (Fig. 1c of Cherniak and Dimanov (2010) and they reported D values for the natural sample in the range of 6.0 × 10^−20^–1.4 × 10^−19^* m*^2^ *s*^−1^ at 1100 °C with c larger than a^*^.

The lack of (a clear) observable diffusion anisotropy in our study might be related to the following issues: (1) Our data reveal an angular deviation of the measured profiles to the closest crystallographic axes of 12.2˚ ± 5.5˚ for the c-axis and of 25.6˚ ± 10.4˚ for the a^*^/b-axis, which might decrease a potentially present anisotropy. (2) The a- and b-axes were treated as one single axis (a^*^/b-axis) for the following reasons: (a) as shown by Zhang et al. (2010), the largest anisotropy was detected between the c-axis and the two other axes that revealed nearly identical D values; and (b) at every *T* at least one experiment with one data point for each axis is required to constrain the Arrhenius equation for this specific axis. This was the case for the data parallel to the c-axis but not perpendicular to it. In any case, anisotropic behavior is, if present at all, rather small and, as stated by Zhang et al. (2010), vanishes with increasing *T*. This observation is in agreement with Dimanov et al. (1996) and Dimanov and Jaoul (1998) who reported isotropic diffusional behavior along b- and c-axis at high *T*. Applying this observation to our results, we conclude that the experimental *T* (1110–1260 °C) of the Di/An system were most likely too high to obtain detectable anisotropy and/or outside of the analytical resolution of the employed instruments.

Our data do not reveal a clear trend regarding *fO*_*2*_ dependence. The highest *T* experiments exhibit a clear positive trend, whereas the lower *T* ones result a slightly negative trend. One potential reason for this could be that the profiles in the high *T* experiment were generally broader and, therefore, the data are more reliable. The investigated *fO*_*2*_ range was rather small (FMQ ± 1) keeping it within the range typically stated for shallow magma chambers. Previous studies revealed divergent results for the dependency on *fO*_*2*_ depending on the investigated cation (which needs to be considered in our case in a coupled form). All studies have in common that any *fO*_*2*_ dependence (positive or negative) was explained by the model of Jaoul and Raterron (1994), which is only valid for Fe-bearing diopsidic cpx (here only very minor Fe in the crystal seeds; Table 3). As our data reveal a minor and inconsistent positive dependence on *fO*_*2*_ for *λ*1 of *m* = 0.021 ± 0.11 (considering only the high *T* experiments we obtain a value of *m* = 0.16 ± 0.13); we favor a cation vacancy-based model (Eq. 12) as the dominant point defect (even if the averaged value of 0.021 is ten times smaller than the proposed value of 3/16 by Jaoul and Raterron (1994)) defined by the following equation to account for the positive *fO*_*2*_ dependence:

12 $$6{\text{Fe}}_{\text{Me}}^{\text{x}} + {\text{Me}}_{\text{Me}}^{\text{x}} + {\text{Si}}_{\text{Si}}^{\text{x}} + \frac{3}{2}{\text{O}}_{2} \left( g \right) \rightleftharpoons {\text{MeSiO}}_{3} + V_{\text{Me}}^{''} + V_{\text{Si}}^{''''} + 6Fe_{\text{Me}}^{^\circ } .$$

The notation follows the work of Jaoul and Raterron (1994) and Kröger (1974) where $X_{Y}^{Z}$ refers to an element *X* (Me (Ca, Mg or Fe), Si, O) or a vacancy (*V*) on a crystallographic site *Y* commonly occupied by element *X*. *Z* is the excess charge relative to the standard cation occupying the respective site where a dot (°) refers to an extra positive and a prime (‘) to an extra negative charge while (^x^) indicates a regular charged site. (*g*) stands for gaseous and *MeSiO*_*3*_ for the pyroxene. The generally smaller and relatively inconsistent fO_*2*_ dependence of our study is most likely related to the following two issues:

(1) We investigated the coupled diffusion of chemical species and not the self-diffusion of single cation so that multiple dependencies (negative or positive) for the different cations and different diffusion mechanisms (cation vacancies and the interstitial defects) are superimposed on each other potentially canceling out and, thus, resulting in an overall smaller dependence. For instance, a negative dependence was shown for ^44^Ca self-diffusion in natural diopsides by Dimanov and Jaoul (1998) who extended the experiments of Dimanov et al. (1996) to higher *T* and variable *fO*_*2*_ conditions (10^−5^–10^−17^ *atm*). Oxygen fugacity dependence was fit with a power exponent of m = − 0.19 ± 0.03 at lower *T* (1100 °C) and revealed a diffusional isotropy at *T* ≥ 1250 °C. Dimanov and Jaoul (1998) proposed an extrinsic interstitial point defect model based on the value of -3/16 by Jaoul and Raterron (1994) that was also used to explain the negative dependence of Si self-diffusion on *fO*_*2*_ by Béjina and Jaoul (1996). As their data were affected by large uncertainties (probably due the employment of a fixed gas mixture to control *fO*_*2*_), they used the theoretically derived value of -3/16. In contrary, a positive *fO*_*2*_ dependency (0.229 ± 0.036) was inferred by Azough and Freer (2000) for ^54^Fe tracer diffusion at a *fO*_*2*_ range between 10^−10^ and 10^−16^* atm*. They likewise proposed a vacancy mechanism as their exponent is close to that calculated by Jaoul and Raterron (1994) of 3/16. Cherniak and Dimanov (2010) compared the activation energies of Fe with the similar value of Mg (150 – 231* kJ mol*^−1^) obtained by Zhang et al. (2010) and suggested that the same vacancy mechanism could be present and, therefore, most likely also a positive *fO*_*2*_ dependence. Nevertheless, this has not been demonstrated unambiguously to date. In summary, only for Ca self-diffusion reliable data have been obtained so far; for Mg the theoretical value of Fe is used and likewise for Si, a theoretical value is inferred. For Al no data are available.

(2) The diopsidic cpx of this study are rather pure containing only small amounts of Fe but as evident from Eq. 12, Fe is required because under oxidizing conditions newly formed diopside exhibits an increased concentration of cation vacancies that are compensated by the oxidation of Fe^2+^ to Fe^3+^. In the present case, where the Fe concentration is basically zero, Jaoul and Raterron (1994) propose that electron holes (h^.^) could substitute for Fe^3+^. Such a substitution can possibly hinder the electronic charge compensation and, therefore, also the formation of cation vacancies leading to a different value for m.

To finally conclude, our data demonstrate a slight positive *fO*_*2*_ dependency and are most likely isotropic. However, we would like to point out that the diffusion coefficients represent upper limit values so that we cannot rule out, that shortcomings related to convolution may have obscured a potential diffusion anisotropy and/or a clearer trend for the *fO*_*2*_ dependency. Furthermore, due to inconsistencies in the *fO*_*2*_ dependence at different *T* (and also the relatively large uncertainties of the data) we abstain from proposing an exact number for the *fO*_*2*_ dependency and formulated the Arrhenius equation without taking into account any *fO*_*2*_ dependence. Additional experiments with longer run durations would be required to confirm the inferred behavior.

### Comparison with previous studies

The interpretation of our results in comparison with previous diffusion studies in Ca-rich cpx requires some caution because previous studies (1) focused either on self-diffusion or inter-diffusion of (single) elements/cations and not on coupled multi-cation diffusion and (2) our data are based on eigenvalues and eigenvectors that were not presented or calculated in previous studies. The formulation of the global Arrhenius equation for the eigenvalues allows a comparison of the activation energy of *λ1* with previous studies: the obtained 114.4 ± 32.8 *kJ mol*^−1^ of the Di/An system is the lowest values so far determined approaching the range of Fe tracer diffusion (161.5 ± 35* kJ mol*^−1^; Azough and Freer (2000)) and Mg self-diffusion parallel to the c-axis (176.2 ± 17.6 *kJ mol*^−1^; Zhang et al. (2010)). For other cations, significantly higher activation energies were obtained with 221 ± 110.0 *kJ mol*^−1^ for Si self-diffusion (Béjina and Jaoul 1996), 272.0 *kJ mol*^−1^ for Al (Jaoul et al. 1991) and variable results for Ca ranging between 265.0 ± 23.0 *kJ mol*^−1^ (Zhang et al. 2010) and 284 ± 10 *kJ mol*^−1^ for T < 1230 °C and 1006 ± 75 *kJ mol*^−1^ for *T* > 1230 °C (Dimanov and Jaoul 1998).

**Fig. 7:**
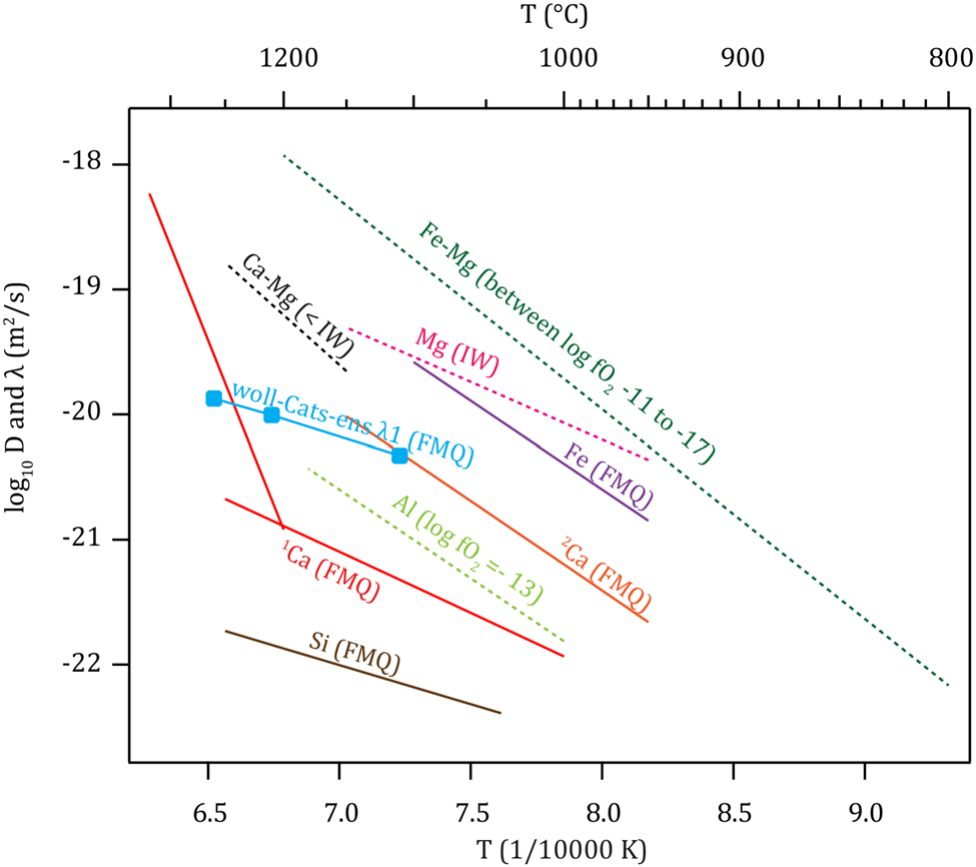
Arrhenius diagram comparing *λ1* from this study (woll-Cats-ens *λ1)* with literature diffusion coefficients. When possible, data were corrected to FMQ and represent diffusion along the c-axis. Solid lines are FMQ corrected values, dotted lines are uncorrected with *fO*_*2*_ conditions indicated in brackets. For Si (Béjina and Jaoul 1996) the theoretical value of -3/16 of Jaoul and Raterron (1994) was used. ^1^Ca is taken from Dimanov and Jaoul (1998) and ^2^Ca from Zhang et al. (2010) with the correction value of Dimanov and Jaoul (1998). Ca–Mg is taken from Brady and McCallister (1983), Fe from Azough and Freer (2000), Mg from Zhang et al. (2010) and Fe–Mg from Müller et al. (2013)

As a direct comparison of our results in the form of D values would require the presentation of all four *D* values (*D*_wol-wol_, *D*_CaTs-wol_, *D*_wol-CaTs_ and D_CaTs-CaTs_), we plot *λ1* in a classical Arrhenius diagram to provide a comparison with previous studies (Fig. 7). If possible, literature data were corrected to FMQ and only c-axis data were used. Our data are situated in the middle of the individual cations composing our system. The *λ1* values plot slightly below Fe tracer diffusion (Azough and Freer 2000) and Mg self-diffusion (Zhang et al. 2010) but above all other cations considered in this study (up to 2.5 orders of magnitude). These results indicate that the coupled diffusion mechanism is obviously not rate limited by the slowest single cation (based on self-diffusing data). Inspecting Fig. 7 in more detail, it reveals that the *λ1* data are lower (in fact would cross them at lower *T*) than the inter-diffusion coefficient of Ca–Mg (Brady and McCallister 1983). Their experiments were not buffered (lack *fO*_*2*_ control and, therefore, uncontrolled point defect concentrations) resulting in activation energies with large uncertainties. Despite this shortcoming, their study is similar to ours in the sense that they investigated multiple cation diffusion that resulted in faster diffusion than the individual self-diffusion data of Ca (Dimanov and Jaoul 1998; Zhang et al. 2010) approaching that of Mg. The same effect is evident for the inter-diffusion of Fe–Mg (Müller et al. 2013) and (Fe, Mn)–Mg (Dimanov and Wiedenbeck 2006) where both inter-diffusion coefficients are higher than the individual self-diffusion of Fe (Azough and Freer 2000) and Mg (Zhang et al. 2010). Obviously, the direct coupling and exchanging of cations (and, therefore, of the species) is faster than self-diffusion which is most likely due to the need of having vacancies or interstitials in the crystal lattice, whereas the coupled mechanism does not exclusively require defects for the diffusion. However, as we detected a slight positive *fO*_*2*_ dependency, defects seem to be still of relevance for the coupled diffusion.

### Geological implications

The key of the experimental results are the diffusion exchange rates characterized by one quarter order of magnitude difference and the corresponding constant eigenvectors. The *T* relationships for *λ1* and *λ2* enable solving for the D matrix using Eq. 2 because the ratios of Eq. 10 and 11 are *T*- and *fO*_*2*_-independent (Fig. A6) and can be treated as constant values. The following equation is used to obtain the D matrix:

13 $$D_{\text{Di/An}} = \left[ {\begin{array}{*{20}c} {1.00} & { - 0.67} \\ { - 0.38} & {1.00} \\ \end{array} } \right]\left[ {\begin{array}{*{20}c} {\lambda 1\left( T \right)} & 0 \\ 0 & {\lambda 2\left( T \right)} \\ \end{array} } \right]\left[ {\begin{array}{*{20}c} {1.00} & { - 0.67} \\ { - 0.38} & {1.00} \\ \end{array} } \right]^{ - 1} ,$$

where $\lambda 1$ and $\lambda 2$ are calculated based on Eqs. 8 and 9. In Table 6, the **D** matrix was calculated for the Di/An system at three different *T* at FMQ using: *λ1*, *λ2, v1* and *v2.*

**Table 6:** Calculation of D matrix at FMQ conditions

T	D × 10^−20^ (m^2^/s)	
1260 °C	1.52	0.52
	− 0.30	0.55
1210 °C	1.13	0.39
	− 0.22	0.40
1110 °C	0.58	0.20
	− 0.11	0.21

Diffusion matrices were calculated based on Eq. 13

Practically, our data can be used to determine the time scales of igneous processes by forward modeling. A common feature in minerals is overgrowth (Dobosi 1989; Simonetti et al. 1996; Morgan et al. 2004; Lierenfeld and Mattsson 2015) attributed to chemical potential changes during crystallization due to magma mixing/assimilation (Gerlach and Grove 1982; Ginibre et al. 2007), ascent towards the surface and/or residence in a second, cooler magma reservoirs at shallower depths inducing decompression-driven crystallization (Sparks and Pinkerton 1978; Blundy and Cashman 2005; Blundy et al. 2006). As the overgrowth event (compositional step function) will be smoothed out by diffusion forming a continuous and (in the ideal case) also broad enough to be measured concentration profile, one can determine the time that elapsed since this event and the quenching of the system (e.g., residence time in magma chamber prior to emplacement and rapid cooling at or near the surface) by fitting the measured profiles to the D value. To date, only inter-diffusion values (e.g., Mg–Fe) have been used for forward modeling (Morgan et al. 2004; Petrone et al. 2016) without considering any complex coupling of the diffusing cations. Thus, the diffusion data presented here should allow a more realistic/appropriate approach to cpx-diffusion chronometry as they simultaneously take into account the coupling of four cations.

#### Example of diffusion modeling of cpx from the Adamello batholith

**Fig. 8:**
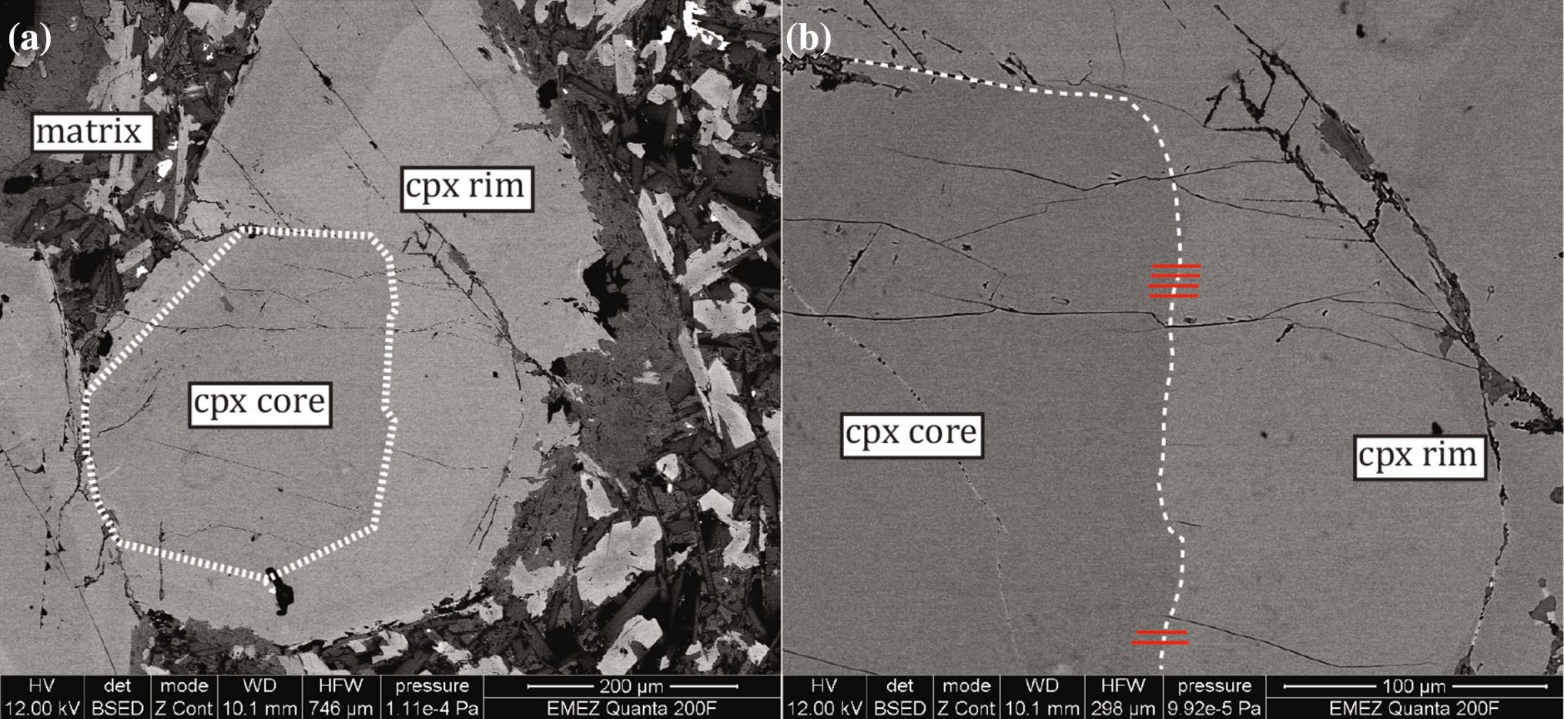
**a** BSE images illustrating the rim/core interface of a natural cpx (augite) from a basaltic dyke rock of the Adamello batholith; **b** a close-up of the location of the six measured profiles

The diffusion data obtained in this study were utilized to quantify the residence time of cpx crystals originating from the well-studied Adamello batholith, Italy, which provides a well-studied “test-field” as the basic geology, petrology and geochemistry were intensively studied both petrographically (Ulmer et al. 1983; Ulmer 1986; Hürlimann et al. 2016) and experimentally (Nimis and Ulmer 1998; Nandedkar et al. 2014). The Adamello batholith is a tertiary calc-alkaline intrusion in the Alps related to an active continental margin. Here we focus on samples from the southernmost superunit (Re di Castello; Callegari and Brack (2002)) that is composed of minor gabbro and diorite and dominant tonalite and subordinate granodiorite. It is crosscut by several generations of post-plutonic dykes covering a wide compositional range from Mg-rich basalt to dacite (Callegari and Brack 2002; Hürlimann et al. 2016). The specific sample investigated (RC171 from Ulmer (1986)) originates from the Monte Re di Castello tonalite unit (for orientation see Fig. 1 of Hürlimann et al. (2016)) specifically from the Bocchetta di Brescia in the vicinity of the summit of the Monte Re di Castello. The sample is classified as basaltic (Ulmer 1986) and composed of pseudomorphosed olivine and fresh cpx, plagioclase and amphibole phenocrysts in a fine-grained matrix consisting of amphibole, plagioclase and magnetite. The cpx phenocrysts exhibit core-rim zoning and were analyzed with the FEG-SEM with the same setup as for the experiments. They reveal near idiomorphic lower-Al, higher-Ca and Mg cores overgrown by higher Al-rims. The obtained profiles (Fig. 8) were treated in the following way: we employed a two-by-two D matrix with the chemical species of the ternary pyroxenoid system wollastonite, Ca-Tschermak and enstatite with the latest being the dependent species. The three species amount to about 85 *mol.*  *%* in the rim and 90 *mol.*  *%* in the core. Not considered for the forward modeling were additional Na- and Fe-bearing species such as jadeite, acmite, esseneite and ferrosilite. For the experiments, the uncertainties were accounted for by computing the standard deviation of the absolute difference between the measured and modeled concentrations. Here, the average of all experiments was determined for each species and subsequently, used as a Gaussian noise added to the measured profiles as a representation of the analytical errors. All computed diffusion times were finally plotted in a histogram and the Monte-Carlo algorithm was repeated for 1000 iterations.

**Fig. 9:**
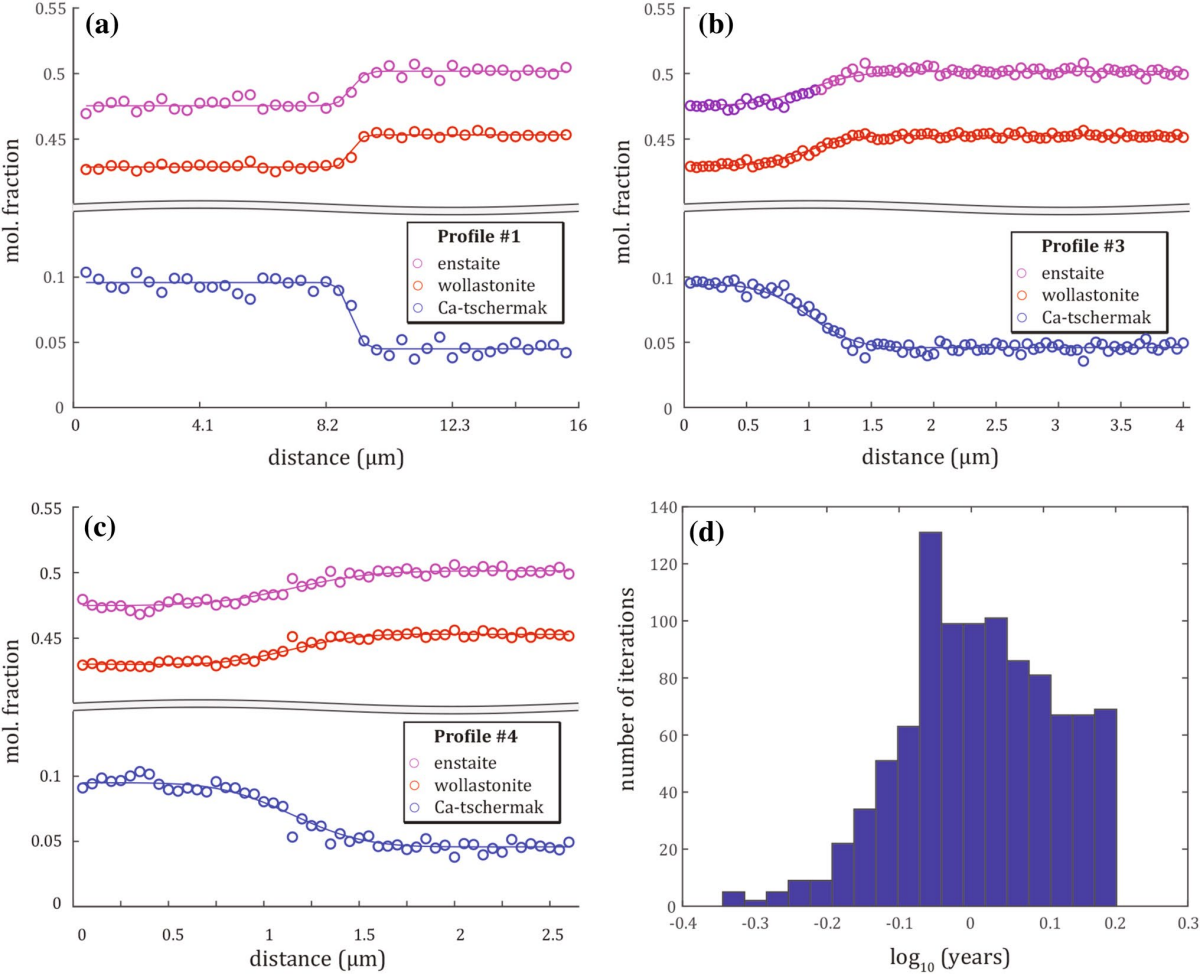
Diffusion modeling of natural cpx phenocrysts at *T* of 970 °C. **a**–**c** Three representative fitted profiles (note variable step-sizes). **d**) Histogram of computed diffusion times for all six profiles (see also Table 7; all original profiles given in Fig. A7)

We used three different *T* conditions (920, 970 and 1020 °C) for the calculation of *λ1* and *λ2* (based on the Eqs. 8 and 9; representing maximum values) with constant *v1* and *v2*. In total, six profiles were measured in a distinctively cored cpx (Fig. 8) characterized by two clear compositional plateaus. All six profiles were modelled simultaneously (non-deconvolved). The resulting timescales vary between 0.68 ± 0.18 to 1.68 ± 0.40 years depending on the applied *T* (Table 7). These data need to be considered as lower limit durations. Figure 9 a – c presents three representative fitted profiles for each step-size for a *T* of 970 °C (all original profiles in Fig. A7).

**Table 7:** Calculated diffusion profiles and best-fit duration of forward modeling of natural Adamello cpx at different *T*

pr.	step-size	1020 °C		970 °C		920 °C	
	(µm)	avg. (yr.)	std.	avg. (yr.)	std.	avg. (yr.)	std.
1	0.41	0.68	0.18	1.05	0.25	1.68	0.40
2	0.10						
3	0.05						
4	0.05						
5	0.05						
6	0.05						

*Yr.* denotes to years and *std.* to standard deviation which bases on the Monte-Carlo simulation (Fig. 9d)

Previous studies on these dyke rocks suggested that the cpx cores crystallized at depths of > 20 *km* (Nimis and Ulmer 1998; Hürlimann et al. 2016) but no specific process was associated with the formation of the overgrowth rims. We would like to emphasis that a magma mixing event is rather unlikely (at least for this particular crystal) because X_Mg_ decreased from rim to core (Table A3) which is a sign for an evolving magma composition rather than a new input of fresh, hot and more mafic magma. This is accompanied by increasing Al contents (Table A3) which has been identified to be typical for fractional crystallization (Nandedkar et al. 2014). Additionally, as relatively long time periods were calculated (1.05 ± 0.25 years for a *T* of 970 °C), the ascent to a shallower magma chamber with a subsequent residence is a more likely event (rim formation already during ascent to the shallower magma chamber or at the time of arrival cannot be excluded a priori). However, we clearly conclude that the rim did not form during the final ascent from (some) depth to the shallow level of emplacement (< 7* km*) because the timescales are too long for such a rapid process which is in the several tenths *cm* *s*^−*1*^–*m* *s*^−*1*^ range (Spera 1984; Klügel et al. 1997; Berghuijs and Mattsson 2013; Lierenfeld and Mattsson 2015) resulting in ascents of hours to days and, therefore, are about 2–3 orders of magnitudes faster. Furthermore, the dykes emplaced in an already cool environment which can be inferred based on the documented fission track ages in zircons at the time of emplacement (Viola 2000) indicating a host rock *T* of < 250 °C (Yamada et al. 1995) promoting immediate quenching as evidenced by the lave-like textures.

Thus, the most likely event recorded by the rim overgrowth and the derived time is a growth and subsequent re-equilibration event in an intermediate crustal magma reservoir during a prolonged (1 year) period where the magma accumulated/ponded prior to final emplacement into the shallow-level batholith. The crystal core probably grew at depth > 25 *km* when the mantle-derived magma stalled in the lowermost crust and/or at the curst-mantle boundary and crystallized and fractionated some olivine and cpx to obtain its present bulk composition (that is not primary but fractionated in the order of 18 *wt.*  *%* olivine and 8 *wt.*  *%* cpx (Hürlimann et al. 2016)). The magma was subsequently transported to shallower depths where it ponded and continue to crystallize with the formation of the rim. The existence of this shallower magma reservoir is inferred from phase relations combined with experimental studies (Nandedkar et al. 2014; Ulmer et al. 2018) indicating that simultaneous saturation of cpx, plagioclase, amphibole and Fe–Ti-oxide occurs around 6–7 *kbar* (20 *km* depth) at 970–1020 °C. The residence time in this shallower magma chamber was in the range of 0.68 ± 0.18 to 1.68 ± 0.40 years. Extraction from this reservoir and ascent to the final emplacement location at a depth of about 5–8 *km* in the already solidified and cool tonalite effectively “froze in” the diffusion.

Our findings infer that (1) the experimentally determined eigenvectors of the diffusion matrix of multicomponent cpx are adaptable to natural samples even if the diffusion couples are not identical to the experimental ones and (2) the data can be employed to model complex cpx diffusion profiles.

## Conclusions

For the first time, the coupled multicomponent diffusion of the pyroxene end-member species wollastonite, Ca-Tschermak and enstatite in diopsidic cpx was investigated by measuring diffusion profiles perpendicular to the diffusion interface. The employed SO technique has some intrinsic problems and we faced partly similar issues as described by Vielzeuf et al. (2007) such as the limited compositional range of the overgrowth rims obtainable (small compositional differences of the diffusion couples) constrained by the anorthite–diopside binary system. The formation of a second generation of rims did not influence our results because all first-generation rims were formed at the identical *T* and sealed off the crystal seeds. The results obtained in this study base on a rather unique combination of different analytical tools. In particular, the application of X-ray *µ*CT and EBSD was of great importance to recover and pre-orient the diopside crystal seeds. The combination of EMPA and EDS in a FEG-SEM step-profiling mode proved to be robust for measuring profiles of a length of several *µm* (only valid with limitations for the short-duration experiments).

The results derived by least-square minimization reveal that *λ1* is a quarter order of magnitude higher than *λ2* and, consequently, represents the dominant diffusional process. For *λ1* the corresponding eigenvector results a constant exchange of $1.00 {\text{Ca}}_{ 2} {\text{Si}}_{ 2} {\text{O}}_{6} + 0.67 {\text{CaAl}}_{ 2} {\text{SiO}}_{6} \rightleftharpoons 1.67 {\text{Mg}}_{ 2} {\text{Si}}_{ 2} {\text{O}}_{6} .$ Due to limitation of the spatial resolution of the employed methods the results reveal a minor time-dependence; thus, only the long-duration experiments were used to calculate the eigenvalues that should be considered as upper limits values of the true D values. The eigenvalues are consistent with Arrhenian relationships with an activation energy of 114.4 ± 32.8* kJ mol*^−1^. The eigenvalues are bracketed by faster Fe tracer (Azough and Freer 2000) and Mg self-diffusion (Zhang et al. 2010) and slower Ca (Dimanov and Jaoul 1998; Zhang et al. 2010), Al (Jaoul et al. 1991) and Si self-diffusion (Béjina and Jaoul 1996). The coupled diffusion reveals near-isotropic diffusional behavior combined with a minor *fO*_*2*_ dependency with an exponent m of 0.021 ± 0.11 for *λ1* that was not taken into account to derive the Arrhenian equation. The D matrix is described by the Arrhenius equations for *λ1* and *λ2* because the exchange ratios *v1* and *v2* can be treated as a constant under the different conditions investigated.

To obtain the full D matrix additional investigation is required employing different combinations of diffusion couple arrangements [e.g., perpendicular (Trial and Spera 1994)]. However, the application of our results to natural, zoned cpx phenocrysts from dyke rocks of the Adamello batholith demonstrates that the derived data and employed models can be utilized to obtain time duration of magmatic processes even if the compositional setup is different from the experimental one underlying their robustness.

## Electronic supplementary material

Below is the link to the electronic supplementary material.

Supplementary material 1 (pdf 1219 kb)
