# Supplementary material for: Species diffusion in clinopyroxene solid solution in the diopside–anorthite system
Source: Contrib Mineral Petrol. 2019 May 13;174(5):46. doi: 10.1007/s00410-019-1571-9 (PMC6515693; doi:10.1007/s00410-019-1571-9)
Supplement: Supplementary file 1 — Supplementary material 1 (pdf 1219 kb) [file 410_2019_1571_MOESM1_ESM.pdf]

# Electronic Supplement

## a. Polishing

X-ray µCT was employed for the following tasks: (1) Localisation of the crystal seeds and (2) pre-orientation of the sample before polishing. For the latter, X-ray µCT scans were studied in detail with the program CTvox© allowing virtual orienting of the sample in 3D to locate suitable crystal axis planes in the diopside crystal seed. Before polishing, the sample was pre-oriented in hot wax (crystalbond) on a holder support matching the chosen plane from the X-ray µCT scans. An easy handling of the sample was warranted by heating up the crystalbond in order to fine-adjust it to match the target positon from the scans followed by pre-polishing with a 1 $\mu m$ silicon carbide polishing paper on the target plane. Subsequently, the pre-polished sample was hot embedded in acrylic epoxy granulate and polishing was continued with a 3 – 1 $\mu m$ diamond paste until the crystal diopside seed was exposed at the surface. The polishing procedure was guided by direct comparison under the microscope with pictures from the X-ray µCT scan to ensure accordance. If a larger orientation deviation was identified, the sample was cut out from the mount and the polishing procedure started again from step one and was repeated until a satisfying result was achieved. The sample was incessantly impregnated (40 % Laromin and 60 % hardener) to minimize mechanical disintegration during polishing. The last step consisted of fine polishing with a 1 – 0.25 $\mu m$ polycrystalline diamond suspension. Fig. A1 provides a comparison between the X-ray µCT picture and the final result after polishing.

## b. Orientation of profiles

As outlined in section ”EBSD and diffusion matrix” an important aspect of the EBSD measurements was to calculate the orientation of each profile in relation to the diopside crystal axes, i.e. the angles between the profile line to the a-, b- and c-axis, respectively. First, the precise position of each profile was determined by comparing several BSE images and the corresponding orientation maps. Subsequently, the BSE picture from the profile was rotated in plane to match best with the pattern quality (IQ) map from the EBSD measurement. An inverse pole figure was calculated with the direction of the profile line as reference axis in the specimen coordinate system (A1 A2 A3; Fig. A2). As the profile lines are always in the plane of the specimen surface, their component towards A3 is zero. Subsequently, the position of the profile was marked on the EBSD map and the equivalent pixels were highlighted in the respective inverse pole figure. From an interactive reading at these pixels, the (hkl) indices of the profile vector could be recorded. Now, the angle between that vector (actually the vector normal to the plane given by (hkl)) and the crystal axes was calculated by the following equation:

$$cos\theta = \frac{d_1 d_2}{sin^2\beta}\left(\frac{h_1 h_2}{a^2} + \frac{k_1 k_2 sin^2\beta}{b^2} + \frac{l_1 l_2}{c^2} - \frac{(l_1 h_2 + l_2 h_1)cos\beta}{ac}\right) \qquad \text{(A1)}$$

with $a$ (9.585 $Å$), b (8.776 $Å$) and $c$ (5.26 $Å$) being the unit cell axes and $\beta$ (106.85°) the unit cell angle for a diopside with monoclinic crystal symmetry. The $h$, $k$, $l$ values with subscript 1 correspond to the profile line as described above, those with subscript 2 are approximate indices of a plane (hkl) perpendicular to one of the crystal axes a, b, c. Specifically, the directions given in [uvw] as a [100], b [010], c [001] are

approximately parallel to plane normal vectors given by (25 0 -4), (010) and (- 9 0 17), respectively. The lattice spacing of the respective planes (hkl) was calculated with the following equation:

$$\frac{1}{d^2} = \frac{1}{sin^2\beta}\left(\frac{h^2}{a^2} + \frac{k^2 sin^2\beta}{b^2} + \frac{l^2}{c^2} - \frac{2hlcos\beta}{ac}\right) \quad \text{(A2)}$$

## c. Additional details of the modelling approach

As described in section "Experimental setup", the diffusion couples were created by applying the SO technique i.e. each couple was produced at the same experimental conditions (3 h and 1310°C; variable $fO_2$) and the diffusion profiles were always measured perpendicular to the rim/core interface. In general, the entire modelling is based on the work of Trial and Spera (1994) as described in section " Data analysis". Due to the isothermal $T$ during the formation of the diffusion couple, the energy equation for the conversation of heat is not required but the conservation of mass. This is given by the following equation which is valid for a constant **D** for each independent chemical species:

$$\frac{\partial w_i}{\partial t} = \sum_{j=1}^{N} D_{ij} \frac{\partial^2 w_j}{\partial x^2} \quad \text{(A3)}$$

Where $w_i$ is the mass fraction, $N$ is the number of independent components, $t$ is the time, $Dij$ are the chemical diffusion coefficients and $x$ the position. **D** is considered constant and in order to uncouple it from its off-diagonal terms, a linear transformation is required to create a new set of chemical components where $\widehat{w}_i$ are the so called eigen-components (Trial and Spera, 1994):

$$\frac{\partial \widehat{w}_i}{\partial t} = \lambda_i \frac{\partial^2 \widehat{w}_i}{\partial x^2} \quad \text{(A4)}$$

$$w_i = \sum_{j=1}^{N} P_{ij} \widehat{w}_j \quad \text{(A5)}$$

$$\boldsymbol{D} = \boldsymbol{P} \Lambda \boldsymbol{P}^{-1} \quad \text{(A6)}$$

The columns of $\boldsymbol{P}$ are the eigenvectors of $\boldsymbol{D}$, $\lambda_i$ are the eigenvalues of $\boldsymbol{D}$ and $\Lambda$ = diag ($\lambda_i$). In this formulation, the eigenvalues ($\lambda_i$) are positive. The solution to equation A3 is the following:

$$w_i = w_i^{bulk} + \sum_{j=1}^{N} \sum_{k=1}^{N} P_{ij} f_i P_{jk}^{-1} \Delta w_k \quad \text{(A7)}$$

where $w_i$ are the modelled concentrations and $\Delta w_k$ are the initial concentration differences across the couple. For short time, the couple is defined as an infinite medium with the following definitions:

$$w_i^{bulk} = \frac{w_i^{(1)} + w_i^{(2)}}{2} \quad \text{(A8)}$$

$$f_i = -\frac{1}{2} \text{erf}\left(\frac{x}{2\sqrt{\lambda_i t}}\right) \quad \text{(A9)}$$

where $w_i^{bulk}$ is the bulk composition of the couple and erf is the error function. A chi-square minimizing approach has been used to minimize the sum of distances between the modelled and measured concentrations by finding the best-fit eigenvalues and eigenvectors of a diffusion matrix where chi-square ($\chi^2$) is minimal:

$$\chi^2 = \sum_{j=1}^{M} \sum_{i=1}^{N+1} \left( \frac{c_i(x_j,t_j) - w_i(\boldsymbol{a};x_j,t_j)}{\sigma_{i,j}} \right)^2 \quad \text{(A10)}$$

where $w_i(\boldsymbol{a};x_j,t_j)$ are the concentrations predicted from the model and $c_i(x_j,t_j)$ are the measured concentrations. As outlined by Trial and Spera (1994), the eigenvalues and eigenvectors were merged into the single vector $\boldsymbol{a}$. $M$ is the number of FEG-SEM measuring points of all profiles obtained at identical temperature and oxygen fugacity and $\sigma_{i,j}$ is the uncertainty of the concentration of component $i$ at ($x_j$, $t_j$). All components were used for the chi-square method ($N+1$) giving a total number of data points of $M(N+1)$. Once the best-fits for the eigenvalues and eigenvectors were found, the diffusion matrix **D** can be computed from equation A6. Additionally, two Taylor series expansion terms were used to approximate (linear and quadratic) the surface of $\chi^2$ expressed by the following equation:

$$\chi^2 \approx \chi_0^2 + D(\chi_0^2)\Delta\boldsymbol{a} + \frac{1}{2}\Delta\boldsymbol{a}^T H(\chi_0^2)\Delta\boldsymbol{a} \quad \text{(A11)}$$

where $D(\chi_0^2)$ is the Jacobian (row vector of the first derivatives) and $H(\chi_0^2)$ is the Hessian (matrix of second derivatives) both evaluated at the parameter $\mathbf{a}^0$ giving the following equations:

$$D(\chi^2)_i = \frac{\partial \chi^2}{\partial a_i} = -2 \sum_{l=1}^{M} \sum_{k=1}^{N+1} \frac{[c_k(x_l,t_l) - w_k(\boldsymbol{a};x_l,t_l)]}{\sigma_{kl}^2} \frac{\partial w_k}{\partial a_i} \quad \text{(A12)}$$

$$H(\chi^2)_{ij} = \frac{\partial^2 \chi^2}{\partial a_i \partial a_j} \approx 2 \sum_{l=1}^{M} \sum_{k=1}^{N+1} \frac{1}{\sigma_{kl}^2} \frac{\partial w_k}{\partial a_i} \frac{\partial w_k}{\partial a_j} \quad \text{(A13)}$$

## d. Rim composition

The crystallization of two generations of cpx grains and overgrowth rims is due to the $T$ reduction step after the 3 h overgrowth rim formation on the seed crystals. At a $T$ of < 1270°C, anorthite co-precipitates ($T < T_{\text{eutectic}}$) and the composition of the newly crystallizing second generation cpx coexisting with anorthite represents partial equilibration with anorthite. The phases from a distinct generation reveal comparable BSE brightness contrast pointing to identical compositions. However, the diffusion couples were not affected by the second generation rim because the first one grew always at the same $T$ (1310°C) and, therefore, compositionally "sealed" off the crystal seed.

In the Di/An system, two main characteristics were repeatedly occurring in the rims (Fig. A4): (1) in the first generation rim, the concentration of Al increases and that of Mg and Si decreases towards the seed crystal; (2) in the second generation rim, the concentration of Al increases towards the contact to the surrounding fine-grained matrix. The first concentration change was possibly promoted by the fact that during the rim growth at 1310°C, a finite amount of diopsidic cpx (< 10 *vol. %*) crystallized. This resulted in a change of the melt composition in the vicinity of these crystals, resulting in an enrichment of the Al content

in the melt phase. Due to the crystallization and the accompanying compositional "corona", the growing rim (first generation) around the diopside crystal seed was affected by these inhomogeneous rim compositions. Beside the corona effects, also mineral inclusion could be a reason for this feature. The profiles characterized by the described feature were discarded for data analysis. The second observed concentration change can be linked to the *T* reduction step (crystallization of additional cpx and anorthite). The second generation rim formed from a eutectic mixture resulting in compositionally different rims in comparison to the first generation rim (Fig. A4). These second generation rims were not always recorded in our profiles. This is possibly because the first rim varies in thickness and the thicker it was the possibility was less of recording the second generation rim. If the profiles in the first generation rims (plateau) were not sufficiently long, these profiles were discarded from further data analysis.

## Reference


Trial AF, Spera FJ (1994) Measuring the multicomponent diffusion matrix: experimental design and data analysis for silicate melts. Geochim Cosmochim Acta 58: 3769-3783

# Figures & Tables

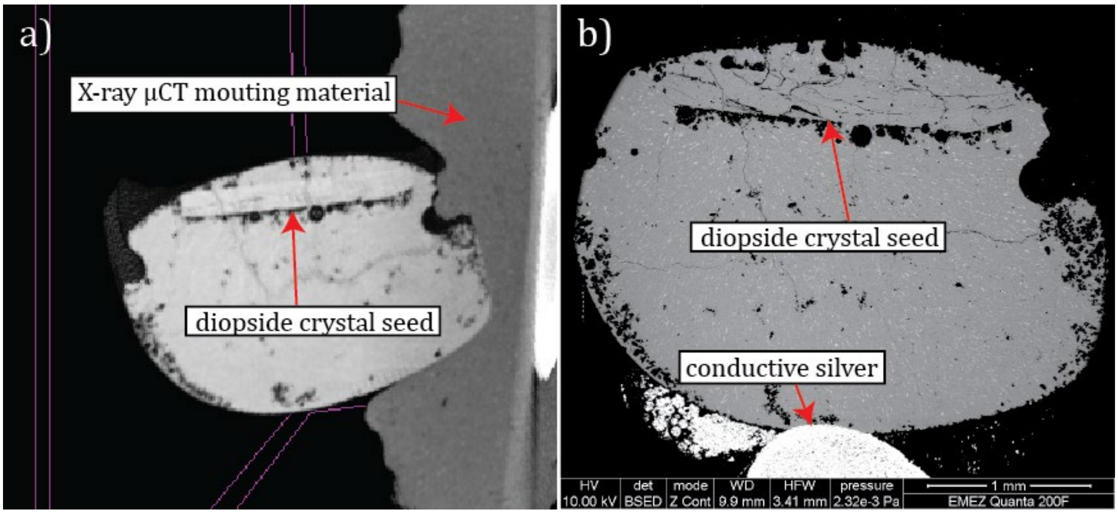


***Fig. A1****: Comparison between* ***a)*** *X-ray µCT scan and* ***b)*** *BSE image (FEG-SEM) of the same sample verifying that the pre-orientation resulted in a satisfying match between the ideal orientation aimed for (a) and the final result after the polishing (b).*

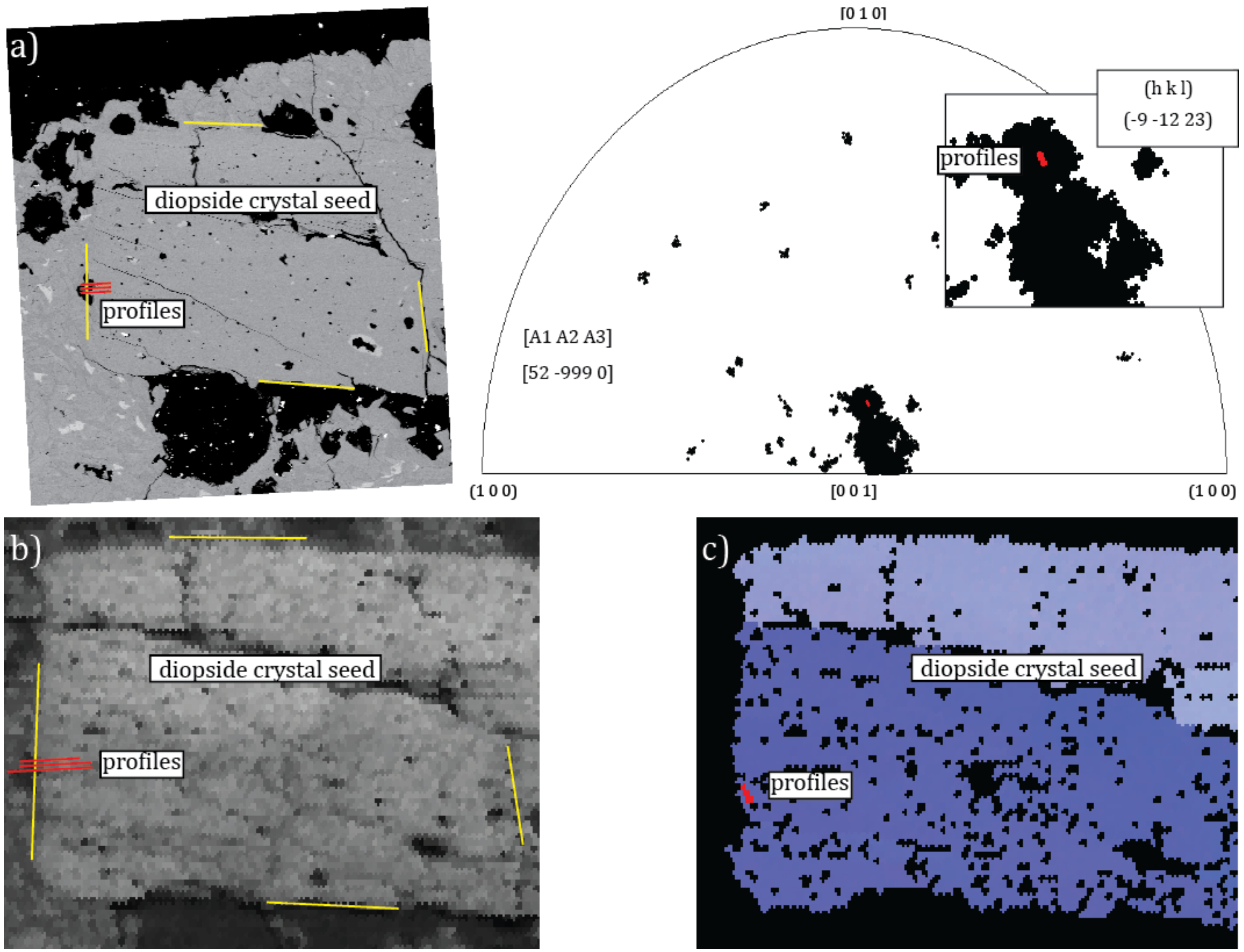


***Fig. A2***: *Schematic drawing illustrating the protocol used to establish the (hkl) indices for each individual profile in order to determine its orientation using equation A1.* ***a)*** *BSE image from the EDS based FEG-SEM profiling measurement with the location of the profiles. The BSE image was rotated for 183° to coincide with the picture from* ***b)*** *which is a pattern quality (IQ) map from the EBSD measurement. The yellow lines represent "guiding" lines used to match the orientation of the crystal. In* ***c)*** *the respective orientation map (using IPF color key) from b) is shown. Some pixels at the location of the three profile locations are highlighted in red "by hand" in the OIM Analysis software. The corresponding crystal orientations are highlighted as well in* ***d)*** *which is an inverse pole figure with the profile line as reference direction in specimen coordinates [A1 A2 A3]. The (hkl) values for the highlighted pixels were extracted interactively from the inverse pole figure using the OIM*

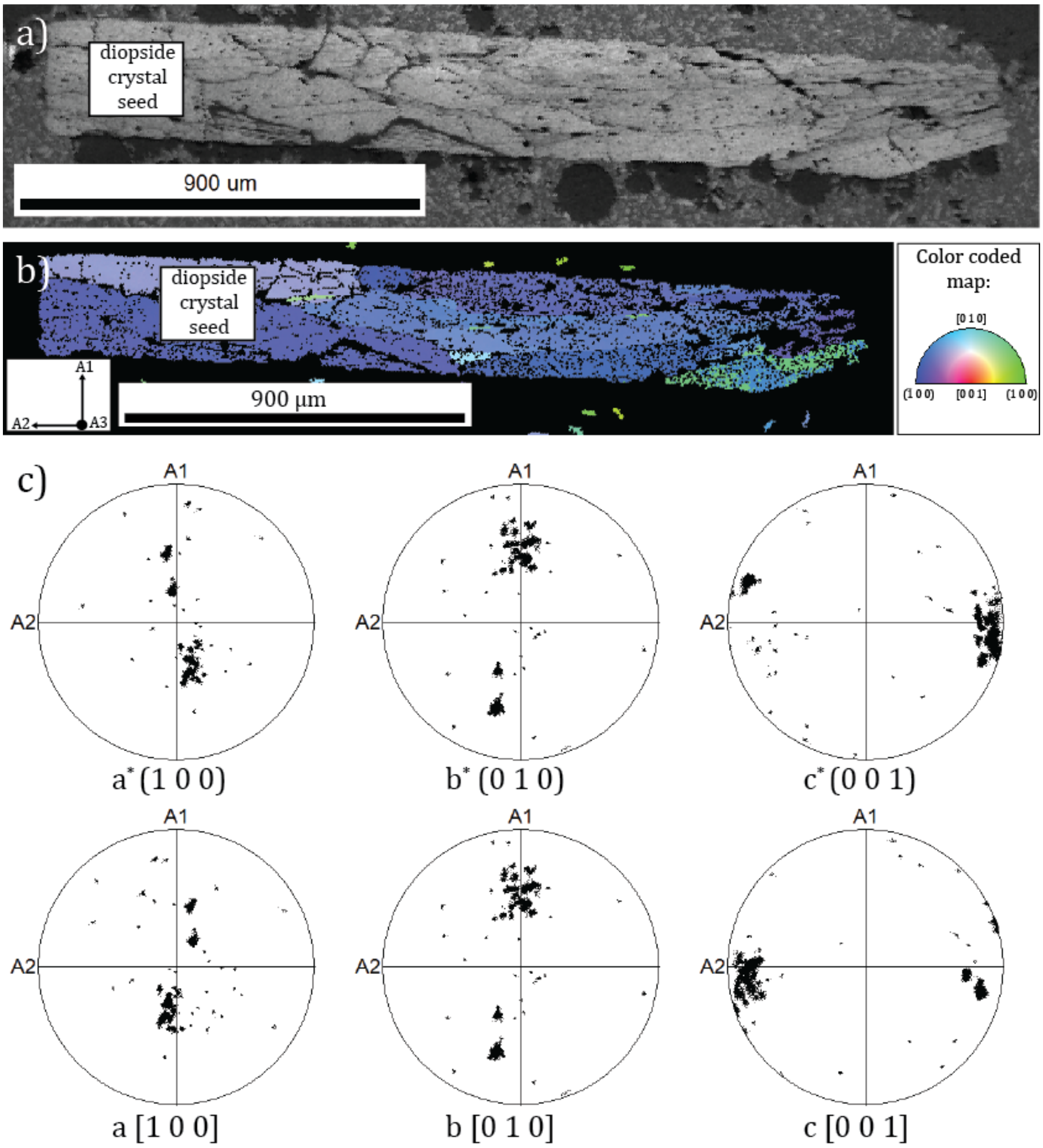


***Fig. A3**: Results of the EBSD measurement of sample # 34 representing a typical diopside crystal seeds exhibiting poly-grain appearance.* ***a)*** *Pattern quality map from the EBSD measurement.* ***b)*** *Orientation map using IPF false color key.* ***c)*** *Corresponding pole figures of the crystal seed for a*, b*, c* planes and for a, b and c axes.*

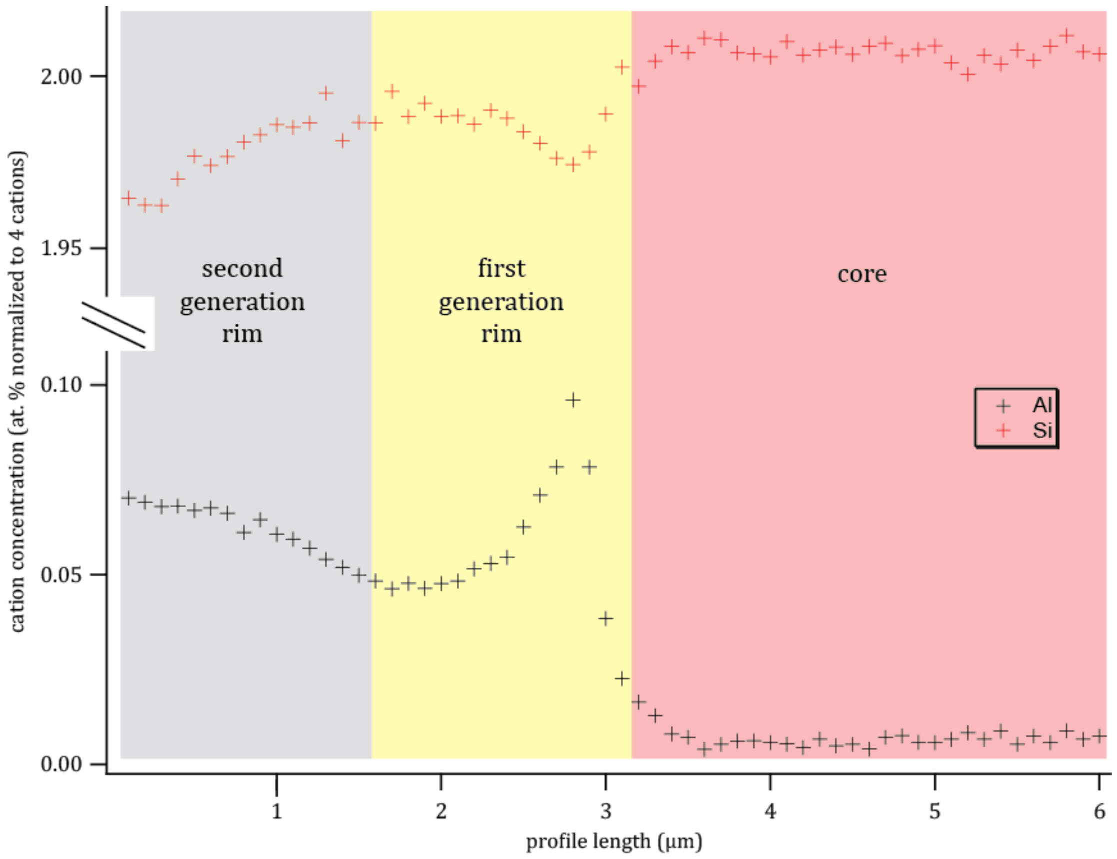


***Fig. A4***: *Deconvoluted cation concentration profile of sample # 34 indicating the change of the concentrations related to the second generation rim and the incomplete "uphill" profile in the first generation rim. We classify it as incomplete because no sign of "uphill" diffusion is detectable in the crystal seed (e.g. decreasing Al concentration). Such profiles were discarded.*

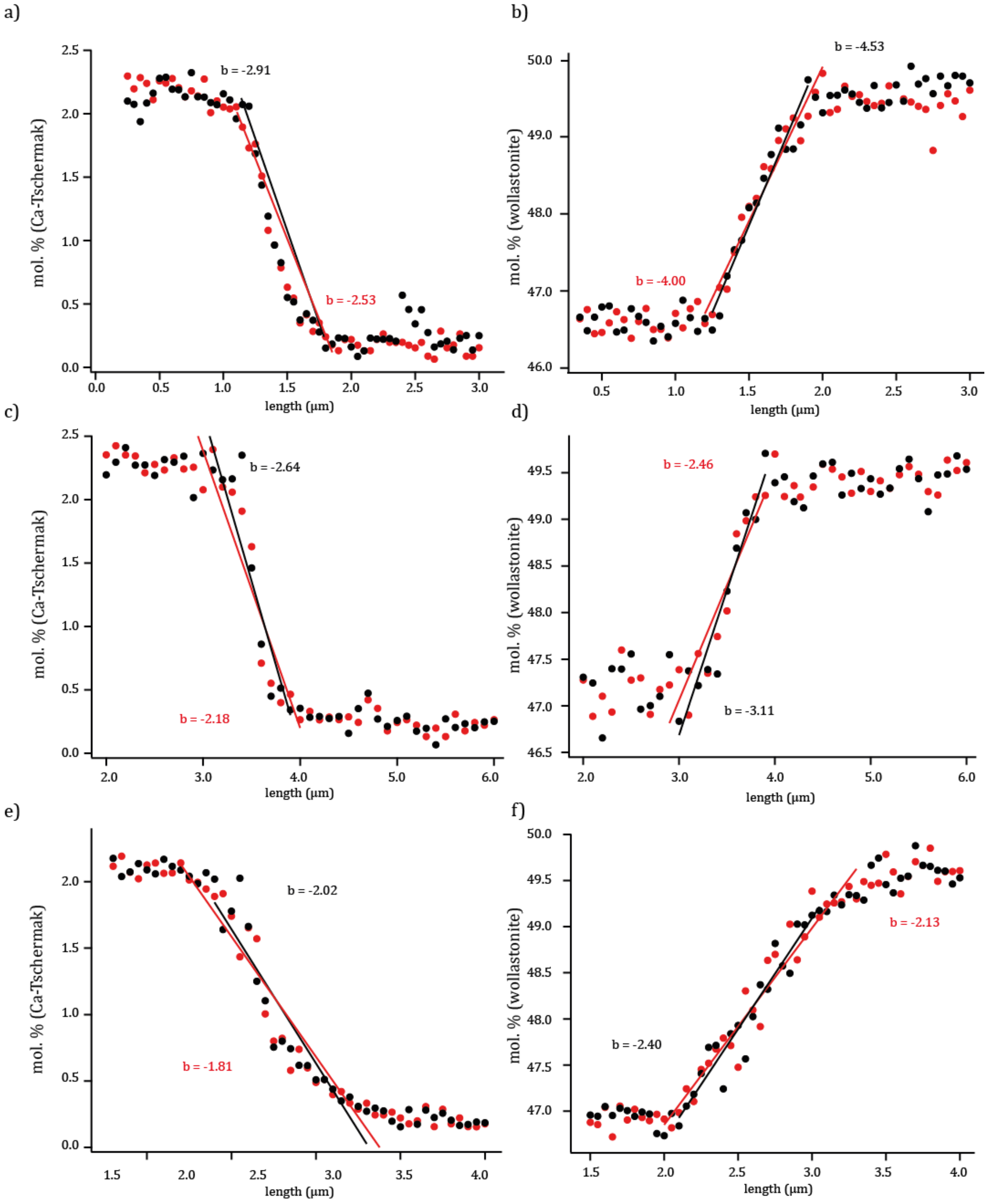


***Fig. A5**: Comparison between non-deconvolved (red) and deconvolved (black) profiles. The slope (m) of the deconvolved profiles is always higher (steeper) than of the non-deconvolved ones. The profiles are from different experiments: **a) and b**) # 23A; **c) and d**) # 30B; and **e) and f**) # 31C.*

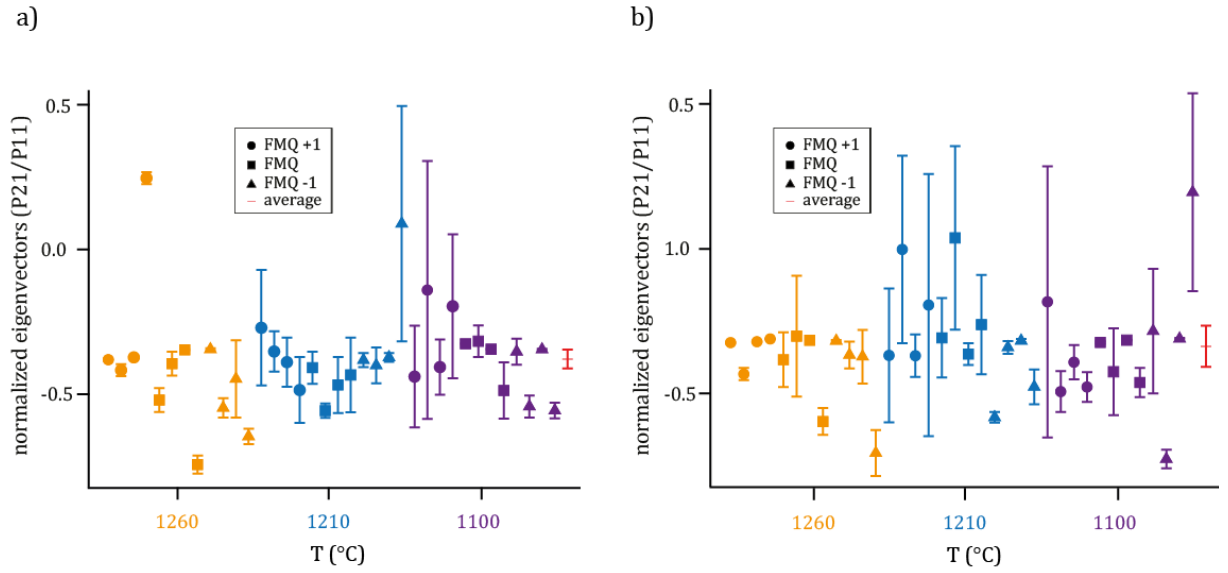


***Fig. A6***: *Eigenvectors of all experiments as a function of experimental T with* ***a)*** *v1 with P21 normalized by P11 and* ***b)*** *v2 with P12 normalized by P22. Circles denote FMQ+1, squares FMQ and triangles FMQ-1 $fO_2$ conditions. Orange symbols indicate experiments conducted at 1260°C, blue at 1210°C and purple at 1110°C and average values are plotted for reference.*

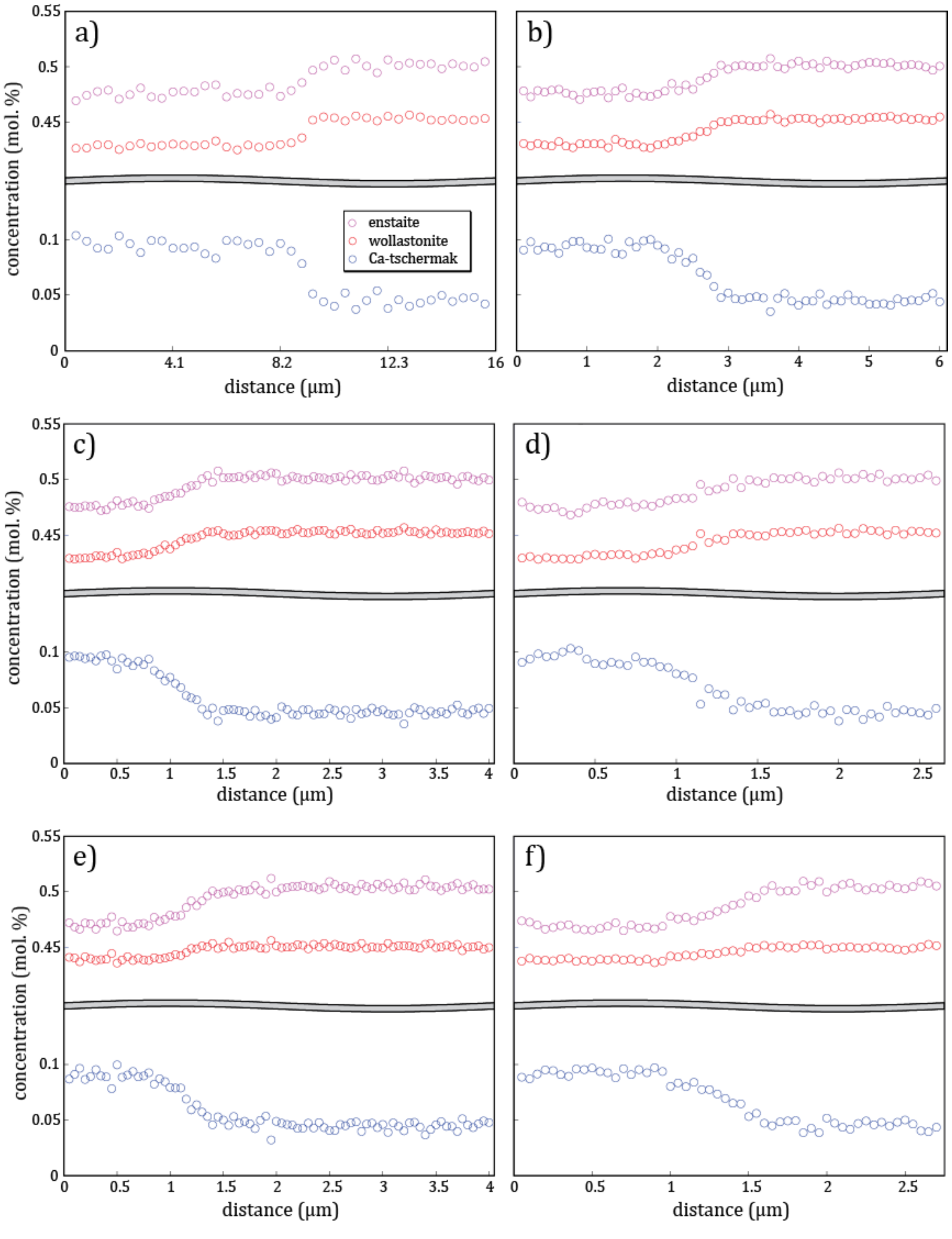


***Fig. A7***: *Original profiles of natural cpx-phenocrysts from a basaltic dyke rock from the Adamello batholith used for the forward modelling.*

***Table A1**: Coefficients used to fit the measured profiles through the crack to the complementary error function of the form:* $a * erfc * [b(x - c)] + d$

| | CaO | MgO | $SiO_2$ | $Al_2O_3$ |
|---|---|---|---|---|
| a (wt. %) | 12.630 | 8.932 | 27.090 | |
| b | 4.204 | 4.400 | 4.346 | |
| c (µm) | 1.069 | 1.029 | 1.039 | |
| d (wt. %) | 0.702 | 0.096 | 0.591 | |
| FWHM (µm) | 0.559 | 0.534 | 0.541 | |

*a denotes the half (concentration) between both ends of the profile; b denotes the steepness of the fitted curve; c denotes the position on the x-axis and d the movement on the y-axis; FWHM is the full width at half maximum which is equivalent to the spatial resolution.*

***Table A2**: EMPA measurements of experimental*

| | # 22.2 | | | | | |
|---|---|---|---|---|---|---|
| | cpx crystal seed | | anorthite | | matrix cpx | |
| | avg. (30) | std. | avg. (32) | std. | avg. (23) | std. |
| $SiO_2$ | 55.54 | 0.21 | 44.75 | 0.10 | 54.59 | 0.34 |
| $Al_2O_3$ | 0.06 | 0.02 | 35.07 | 0.09 | 1.68 | 0.48 |
| FeO | 0.14 | 0.07 | 0.07 | 0.02 | 0.11 | 0.03 |
| MgO | 18.34 | 0.10 | 0.65 | 0.03 | 18.55 | 0.33 |
| CaO | 26.07 | 0.10 | 19.84 | 0.10 | 25.04 | 0.22 |
| $Na_2O$ | 0.02 | 0.01 | 0.27 | 0.02 | 0.01 | 0.01 |
| Total | 100.16 | 0.17 | 100.65 | 0.14 | 99.98 | 0.23 |

| | # 29 | | | | | |
|---|---|---|---|---|---|---|
| | cpx crystal seed | | anorthite | | matrix cpx | |
| | avg. (20) | std. | avg. (19) | std. | avg. (6) | std. |
| $SiO_2$ | 55.23 | 0.15 | 44.37 | 0.26 | 54.12 | 0.44 |
| $Al_2O_3$ | 0.18 | 0.19 | 34.02 | 0.56 | 2.53 | 0.81 |
| FeO | 0.10 | 0.03 | 0.02 | 0.01 | 0.13 | 0.02 |
| MgO | 18.66 | 0.12 | 0.72 | 0.20 | 18.75 | 0.28 |
| CaO | 26.02 | 0.20 | 20.07 | 0.18 | 24.88 | 0.28 |
| $Na_2O$ | 0.01 | 0.01 | 0.16 | 0.03 | 0.01 | 0.01 |
| Total | 100.19 | 0.20 | 99.36 | 0.33 | 100.41 | 0.27 |

| | # 29 | | | |
|---|---|---|---|---|
| | cpx crystal seed | | anorthite | |
| | avg. (30) | std. | avg. (32) | std. |
| $SiO_2$ | 55.27 | 0.18 | 44.55 | 0.12 |
| $Al_2O_3$ | 0.13 | 0.13 | 35.09 | 0.17 |
| FeO | 0.07 | 0.02 | 0.02 | 0.01 |
| MgO | 18.67 | 0.11 | 0.69 | 0.03 |
| CaO | 25.88 | 0.14 | 20.05 | 0.11 |
| $Na_2O$ | 0.02 | 0.02 | 0.17 | 0.02 |
| Total | 100.04 | 0.10 | 100.57 | 0.27 |

| | # 23 | | | | | | | |
|---|---|---|---|---|---|---|---|---|
| | cpx crystal seed | | anorthite | | matrix cpx | | wollastonite | |
| | avg. (30) | std. | avg. (32) | std. | avg. (24) | std. | avg. (18) | std. |
| $SiO_2$ | 55.40 | 0.14 | 44.29 | 0.26 | 54.45 | 0.48 | 51.75 | 0.11 |
| $Al_2O_3$ | 0.06 | 0.04 | 35.19 | 0.42 | 1.89 | 0.74 | 0.05 | 0.01 |
| FeO | 0.20 | 0.16 | 0.05 | 0.02 | 0.09 | 0.03 | 0.07 | 0.03 |
| MgO | 18.39 | 0.17 | 0.51 | 0.08 | 18.67 | 0.28 | 1.89 | 0.06 |
| CaO | 25.93 | 0.11 | 19.88 | 0.18 | 24.73 | 0.21 | 45.65 | 0.17 |
| $Na_2O$ | 0.02 | 0.02 | 0.13 | 0.02 | 0.01 | 0.01 | 0.01 | 0.01 |
| Total | 99.99 | 0.19 | 100.05 | 0.30 | 99.84 | 0.28 | 99.41 | 0.21 |

| | # 30 | | | | | |
|---|---|---|---|---|---|---|
| | cpx crystal seed | | anorthite | | matrix cpx | |
| | avg. (30) | std. | avg. (27) | std. | avg. (18) | std. |
| $SiO_2$ | 54.98 | 0.15 | 43.92 | 0.43 | 53.86 | 0.42 |
| $Al_2O_3$ | 0.04 | 0.03 | 34.91 | 0.57 | 2.45 | 0.54 |
| FeO | 0.10 | 0.07 | 0.05 | 0.02 | 0.12 | 0.03 |
| MgO | 18.65 | 0.11 | 0.56 | 0.18 | 18.60 | 0.23 |
| CaO | 25.81 | 0.14 | 19.91 | 0.15 | 24.71 | 0.12 |
| $Na_2O$ | 0.01 | 0.01 | 0.17 | 0.04 | 0.01 | 0.01 |
| Total | 99.59 | 0.25 | 99.52 | 0.28 | 99.75 | 0.21 |

***Table A2*** *continued.*

| | # 25.2 | | | | | |
|---|---|---|---|---|---|---|
| | cpx crystal seed | | anorthite | | matrix cpx | |
| | avg. (30) | std. | avg. (30) | std. | avg. (24) | std. |
| $SiO_2$ | 55.55 | 0.15 | 48.27 | 0.41 | 54.71 | 0.23 |
| $Al_2O_3$ | 0.03 | 0.02 | 29.90 | 0.90 | 1.60 | 0.31 |
| FeO | 0.13 | 0.07 | 0.02 | 0.02 | 0.10 | 0.02 |
| MgO | 18.31 | 0.10 | 1.80 | 0.31 | 18.60 | 0.14 |
| CaO | 25.88 | 0.12 | 20.20 | 0.54 | 24.74 | 0.12 |
| $Na_2O$ | 0.02 | 0.01 | 0.14 | 0.03 | 0.00 | 0.00 |
| Total | 99.92 | 0.20 | 100.51 | 0.26 | 99.75 | 0.23 |
| | **# 32** | | | | | |
| | cpx crystal seed | | anorthite | | matrix cpx | |
| | avg. (30) | std. | avg. (21) | std. | avg. (24) | std. |
| $SiO_2$ | 55.60 | 0.16 | 47.96 | 0.50 | 53.75 | 0.70 |
| $Al_2O_3$ | 0.05 | 0.03 | 30.57 | 0.69 | 3.06 | 0.99 |
| FeO | 0.10 | 0.04 | 0.20 | 0.03 | 0.13 | 0.04 |
| MgO | 18.43 | 0.09 | 0.91 | 0.09 | 18.02 | 0.59 |
| CaO | 26.14 | 0.13 | 18.92 | 0.40 | 24.95 | 0.24 |
| $Na_2O$ | 0.03 | 0.02 | 0.55 | 0.22 | 0.01 | 0.01 |
| Total | 100.34 | 0.26 | 99.10 | 0.40 | 99.91 | 0.54 |
| | **# 32** | | | | | |
| | cpx crystal seed | | anorthite | | | |
| | avg. (30) | std. | avg. (22) | std. | | |
| $SiO_2$ | 55.50 | 0.16 | 48.12 | 0.41 | | |
| $Al_2O_3$ | 0.04 | 0.02 | 31.12 | 0.39 | | |
| FeO | 0.13 | 0.03 | 0.22 | 0.03 | | |
| MgO | 18.46 | 0.09 | 0.93 | 0.18 | | |
| CaO | 25.99 | 0.11 | 19.23 | 0.34 | | |
| $Na_2O$ | 0.02 | 0.01 | 0.41 | 0.06 | | |
| Total | 100.15 | 0.12 | 100.03 | 0.25 | | |

***Table A2** continued.*

| | # 12 | | | | | |
|---|---|---|---|---|---|---|
| | cpx crystal seed | | matrix cpx | | residual melt | |
| | avg. (28) | std. | avg. (22) | std. | avg. (22) | std. |
| $SiO_2$ | 55.40 | 0.13 | 54.62 | 0.13 | 51.22 | 0.11 |
| $Al_2O_3$ | 0.03 | 0.03 | 1.16 | 0.07 | 11.75 | 0.09 |
| FeO | 0.03 | 0.02 | 0.05 | 0.02 | 0.11 | 0.02 |
| MgO | 18.49 | 0.11 | 18.75 | 0.11 | 12.01 | 0.11 |
| CaO | 26.09 | 0.12 | 24.82 | 0.14 | 24.33 | 0.10 |
| $Na_2O$ | 0.02 | 0.03 | 0.01 | 0.01 | 0.10 | 0.02 |
| Total | 100.05 | 0.20 | 99.40 | 0.18 | 99.53 | 0.19 |
| | **# 13** | | | | | |
| | cpx crystal seed | | matrix cpx | | residual melt | |
| | avg. (29) | std. | avg. (21) | std. | avg. (24) | std. |
| $SiO_2$ | 55.09 | 0.13 | 54.42 | 0.20 | 51.06 | 0.58 |
| $Al_2O_3$ | 0.04 | 0.02 | 1.21 | 0.09 | 11.17 | 1.87 |
| FeO | 0.16 | 0.09 | 0.08 | 0.01 | 0.17 | 0.02 |
| MgO | 18.33 | 0.11 | 18.70 | 0.08 | 12.66 | 1.23 |
| CaO | 25.88 | 0.10 | 24.73 | 0.10 | 24.22 | 0.17 |
| $Na_2O$ | 0.02 | 0.02 | 0.01 | 0.01 | 0.08 | 0.02 |
| Total | 99.52 | 0.15 | 99.16 | 0.18 | 99.36 | 0.19 |
| | **# 14** | | | | | |
| | cpx crystal seed | | anorthite | | matrix cpx | |
| | avg. (30) | std. | avg. (40) | std. | avg. (32) | std. |
| $SiO_2$ | 55.35 | 0.20 | 44.58 | 0.35 | 54.56 | 0.29 |
| $Al_2O_3$ | 0.02 | 0.02 | 35.41 | 0.37 | 1.66 | 0.44 |
| FeO | 0.03 | 0.02 | 0.02 | 0.01 | 0.08 | 0.02 |
| MgO | 18.48 | 0.10 | 0.49 | 0.10 | 18.79 | 0.20 |
| CaO | 26.02 | 0.13 | 19.77 | 0.16 | 24.74 | 0.23 |
| $Na_2O$ | 0.01 | 0.01 | 0.26 | 0.06 | 0.01 | 0.01 |
| Total | 99.92 | 0.16 | 100.53 | 0.15 | 99.83 | 0.20 |
| | **# 27** | | | | | |
| | cpx crystal seed | | anorthite | | matrix cpx | |
| | avg. (30) | std. | avg. (30) | std. | avg. (27) | std. |
| $SiO_2$ | 55.22 | 0.22 | 44.11 | 0.29 | 53.98 | 0.33 |
| $Al_2O_3$ | 0.06 | 0.02 | 35.86 | 0.23 | 1.76 | 0.36 |
| FeO | 0.07 | 0.02 | 0.05 | 0.02 | 0.07 | 0.02 |
| MgO | 18.21 | 0.10 | 0.54 | 0.06 | 18.67 | 0.31 |
| CaO | 25.94 | 0.11 | 20.13 | 0.14 | 24.87 | 0.29 |
| $Na_2O$ | 0.02 | 0.01 | 0.10 | 0.03 | 0.01 | 0.01 |
| Total | 99.53 | 0.26 | 100.73 | 0.18 | 99.36 | 0.25 |

| | # 15.2 | | | | | | | |
|---|---|---|---|---|---|---|---|---|
| | cpx crystal seed | | anorthite | | matrix cpx | | wollastonite | |
| | avg. (30) | std. | avg. (32) | std. | avg. (24) | std. | avg. (12) | std. |
| $SiO_2$ | 55.35 | 0.11 | 44.46 | 0.23 | 54.77 | 0.25 | 51.37 | 0.18 |
| $Al_2O_3$ | 0.05 | 0.03 | 35.20 | 0.40 | 1.57 | 0.37 | 0.24 | 0.31 |
| FeO | 0.11 | 0.03 | 0.13 | 0.02 | 0.10 | 0.02 | 0.16 | 0.01 |
| MgO | 18.56 | 0.09 | 0.56 | 0.11 | 18.89 | 0.18 | 1.90 | 0.07 |
| CaO | 25.96 | 0.13 | 20.12 | 0.20 | 24.78 | 0.17 | 45.27 | 0.36 |
| $Na_2O$ | 0.02 | 0.01 | 0.18 | 0.03 | 0.01 | 0.01 | 0.01 | 0.01 |
| Total | 100.05 | 0.14 | 100.64 | 0.14 | 100.12 | 0.15 | 98.94 | 0.28 |

***Table A2*** *continued.*

| | # 28 | | | | | | | |
|---|---|---|---|---|---|---|---|---|
| | cpx crystal seed | | anorthite | | matrix cpx | | wollastonite | |
| | avg. (19) | std. | avg. (10) | std. | avg. (15) | std. | avg. (9) | std. |
| $SiO_2$ | 55.58 | 0.22 | 44.81 | 0.36 | 54.51 | 0.73 | 51.59 | 0.15 |
| $Al_2O_3$ | 0.05 | 0.03 | 33.33 | 0.97 | 2.33 | 0.96 | 0.11 | 0.03 |
| FeO | 0.08 | 0.04 | 0.01 | 0.01 | 0.05 | 0.02 | 0.02 | 0.02 |
| MgO | 18.71 | 0.09 | 0.67 | 0.13 | 18.52 | 0.69 | 2.01 | 0.03 |
| CaO | 26.18 | 0.14 | 20.17 | 0.34 | 25.22 | 0.49 | 46.16 | 0.13 |
| $Na_2O$ | 0.01 | 0.01 | 0.21 | 0.03 | 0.01 | 0.01 | 0.00 | 0.00 |
| Total | 100.59 | 0.25 | 99.19 | 0.25 | 100.64 | 0.29 | 99.89 | 0.25 |

| | # 28 | | | |
|---|---|---|---|---|
| | cpx crystal seed | | anorthite | |
| | avg. (28) | std. | avg. (37) | std. |
| $SiO_2$ | 55.37 | 0.11 | 44.49 | 0.49 |
| $Al_2O_3$ | 0.07 | 0.04 | 34.90 | 0.48 |
| FeO | 0.06 | 0.02 | 0.01 | 0.01 |
| MgO | 18.68 | 0.10 | 0.52 | 0.13 |
| CaO | 25.78 | 0.11 | 19.77 | 0.14 |
| $Na_2O$ | 0.02 | 0.02 | 0.19 | 0.04 |
| Total | 99.97 | 0.18 | 99.88 | 0.24 |

| | # 24.2 | | | | | |
|---|---|---|---|---|---|---|
| | cpx crystal seed | | anorthite | | matrix cpx | |
| | avg. (30) | std. | avg. (38) | std. | avg. (24) | std. |
| $SiO_2$ | 55.42 | 0.12 | 48.41 | 0.38 | 54.49 | 0.23 |
| $Al_2O_3$ | 0.04 | 0.03 | 30.66 | 1.00 | 1.69 | 0.31 |
| FeO | 0.11 | 0.03 | 0.18 | 0.02 | 0.08 | 0.02 |
| MgO | 18.33 | 0.08 | 1.15 | 0.15 | 18.82 | 0.17 |
| CaO | 25.78 | 0.10 | 19.65 | 0.66 | 24.49 | 0.24 |
| $Na_2O$ | 0.02 | 0.02 | 0.23 | 0.03 | 0.01 | 0.01 |
| Total | 99.70 | 0.16 | 100.28 | 0.30 | 99.58 | 0.27 |

| | # 33 | | | | | |
|---|---|---|---|---|---|---|
| | cpx crystal seed | | anorthite | | matrix cpx | |
| | avg. (20) | std. | avg. (24) | std. | avg. (24) | std. |
| $SiO_2$ | 55.48 | 0.28 | 47.91 | 0.62 | 54.51 | 0.41 |
| $Al_2O_3$ | 0.08 | 0.03 | 29.52 | 0.97 | 1.92 | 0.34 |
| FeO | 0.15 | 0.08 | 0.14 | 0.02 | 0.07 | 0.03 |
| MgO | 18.51 | 0.10 | 0.97 | 0.26 | 18.84 | 0.28 |
| CaO | 26.24 | 0.21 | 19.82 | 1.00 | 24.93 | 0.26 |
| $Na_2O$ | 0.03 | 0.01 | 0.70 | 0.14 | 0.01 | 0.01 |
| Total | 100.48 | 0.30 | 99.06 | 0.48 | 100.28 | 0.34 |

| | # 33 | | | |
|---|---|---|---|---|
| | cpx crystal seed | | anorthite | |
| | avg. (30) | std. | avg. (23) | std. |
| $SiO_2$ | 55.36 | 0.11 | 48.40 | 1.17 |
| $Al_2O_3$ | 0.07 | 0.04 | 30.90 | 0.88 |
| FeO | 0.08 | 0.03 | 0.13 | 0.03 |
| MgO | 18.42 | 0.08 | 0.93 | 0.14 |
| CaO | 26.08 | 0.12 | 19.10 | 0.52 |
| $Na_2O$ | 0.04 | 0.02 | 0.22 | 0.03 |
| Total | 100.04 | 0.12 | 99.68 | 0.31 |

***Table A2*** *continued.*

| | # 20 | | | | | |
|---|---|---|---|---|---|---|
| | cpx crystal seed | | anorthite | | matrix cpx | |
| | avg. (30) | std. | avg. (32) | std. | avg. (20) | std. |
| $SiO_2$ | 55.38 | 0.20 | 44.59 | 0.17 | 53.60 | 0.81 |
| $Al_2O_3$ | 0.10 | 0.07 | 35.26 | 0.13 | 2.73 | 1.09 |
| FeO | 0.17 | 0.08 | 0.01 | 0.01 | 0.07 | 0.02 |
| MgO | 18.27 | 0.14 | 0.64 | 0.02 | 18.27 | 0.53 |
| CaO | 25.75 | 0.21 | 19.81 | 0.09 | 24.61 | 0.29 |
| $Na_2O$ | 0.02 | 0.05 | 0.14 | 0.01 | 0.01 | 0.01 |
| Total | 99.70 | 0.23 | 100.44 | 0.23 | 99.29 | 0.25 |
| | # 31 | | | | | |
| | cpx crystal seed | | anorthite | | matrix cpx | |
| | avg. (30) | std. | avg. (29) | std. | avg. (28) | std. |
| $SiO_2$ | 55.26 | 0.17 | 44.70 | 0.17 | 54.11 | 0.30 |
| $Al_2O_3$ | 0.07 | 0.04 | 35.18 | 0.13 | 2.56 | 0.38 |
| FeO | 0.15 | 0.09 | 0.01 | 0.01 | 0.06 | 0.03 |
| MgO | 18.60 | 0.12 | 0.48 | 0.02 | 18.51 | 0.30 |
| CaO | 25.75 | 0.12 | 19.46 | 0.10 | 24.69 | 0.31 |
| $Na_2O$ | 0.01 | 0.01 | 0.39 | 0.02 | 0.02 | 0.01 |
| Total | 99.83 | 0.29 | 100.22 | 0.28 | 99.94 | 0.27 |
| | # 21 | | | | | |
| | cpx crystal seed | | anorthite | | matrix cpx | |
| | avg. (30) | std. | avg. (32) | std. | avg. (23) | std. |
| $SiO_2$ | 55.25 | 0.10 | 44.39 | 0.33 | 54.52 | 0.35 |
| $Al_2O_3$ | 0.09 | 0.05 | 35.33 | 0.41 | 1.98 | 0.65 |
| FeO | 0.01 | 0.01 | 0.02 | 0.01 | 0.09 | 0.05 |
| MgO | 18.57 | 0.12 | 0.54 | 0.10 | 18.40 | 0.64 |
| CaO | 25.76 | 0.09 | 19.92 | 0.14 | 24.79 | 0.38 |
| $Na_2O$ | 0.04 | 0.02 | 0.12 | 0.02 | 0.01 | 0.01 |
| Total | 99.72 | 0.19 | 100.32 | 0.25 | 99.79 | 0.27 |
| | # 34 | | | | | |
| | cpx crystal seed | | anorthite | | matrix cpx | |
| | avg. (20) | std. | avg. (23) | std. | avg. (24) | std. |
| $SiO_2$ | 55.43 | 0.13 | 44.43 | 0.24 | 54.24 | 0.25 |
| $Al_2O_3$ | 0.04 | 0.02 | 34.08 | 0.36 | 2.08 | 0.39 |
| FeO | 0.10 | 0.04 | 0.03 | 0.02 | 0.17 | 0.10 |
| MgO | 18.51 | 0.12 | 0.41 | 0.07 | 18.40 | 0.39 |
| CaO | 26.25 | 0.13 | 19.89 | 0.20 | 25.23 | 0.43 |
| $Na_2O$ | 0.01 | 0.01 | 0.35 | 0.04 | 0.02 | 0.01 |
| Total | 100.34 | 0.20 | 99.19 | 0.53 | 100.14 | 0.34 |
| | # 34 | | | | | |
| | cpx crystal seed | | anorthite | | | |
| | avg. (29) | std. | avg. (22) | std. | | |
| $SiO_2$ | 55.46 | 0.12 | 44.41 | 0.22 | | |
| $Al_2O_3$ | 0.03 | 0.01 | 35.37 | 0.30 | | |
| FeO | 0.09 | 0.03 | 0.03 | 0.02 | | |
| MgO | 18.47 | 0.10 | 0.42 | 0.07 | | |
| CaO | 26.13 | 0.12 | 19.79 | 0.11 | | |
| $Na_2O$ | 0.01 | 0.01 | 0.31 | 0.04 | | |
| Total | 100.19 | 0.14 | 100.33 | 0.16 | | |

***Table A2** continued.*

| | # 26 | | | | | |
|---|---|---|---|---|---|---|
| | cpx crystal seed | | anorthite | | matrix cpx | |
| | avg. (54) | std. | avg. (59) | std. | avg. (27) | std. |
| $SiO_2$ | 55.51 | 0.14 | 45.28 | 0.62 | 54.56 | 0.51 |
| $Al_2O_3$ | 0.08 | 0.06 | 34.34 | 0.59 | 2.10 | 0.80 |
| FeO | 0.06 | 0.03 | 0.05 | 0.02 | 0.09 | 0.03 |
| MgO | 18.19 | 0.11 | 0.49 | 0.08 | 18.38 | 0.50 |
| CaO | 25.89 | 0.13 | 19.71 | 0.39 | 24.57 | 0.25 |
| $Na_2O$ | - | - | - | - | - | - |
| Total | 99.72 | 0.19 | 99.87 | 0.31 | 99.69 | 0.18 |

| | # 26 | | | |
|---|---|---|---|---|
| | cpx crystal seed | | anorthite | |
| | avg. (28) | std. | avg. (30) | std. |
| $SiO_2$ | 55.12 | 0.15 | 45.11 | 0.37 |
| $Al_2O_3$ | 0.14 | 0.38 | 34.13 | 0.71 |
| FeO | 0.03 | 0.02 | 0.05 | 0.02 |
| MgO | 18.26 | 0.32 | 0.53 | 0.06 |
| CaO | 25.86 | 0.08 | 19.96 | 0.51 |
| $Na_2O$ | 0.05 | 0.07 | 0.22 | 0.04 |
| Total | 99.46 | 0.22 | 100.00 | 0.58 |

| | # 35 | | | | | | | |
|---|---|---|---|---|---|---|---|---|
| | cpx crystal seed | | anorthite | | matrix cpx | | wollastonite | |
| | avg. (30) | std. | avg. (46) | std. | avg. (20) | std. | avg. (5) | std. |
| $SiO_2$ | 55.39 | 0.14 | 48.05 | 0.66 | 54.19 | 0.30 | 51.55 | 0.17 |
| $Al_2O_3$ | 0.04 | 0.04 | 30.76 | 1.52 | 2.47 | 0.50 | 1.12 | 0.43 |
| FeO | 0.08 | 0.02 | 0.10 | 0.05 | 0.08 | 0.02 | 1.22 | 0.04 |
| MgO | 18.57 | 0.11 | 1.32 | 0.84 | 18.43 | 0.32 | 1.12 | 0.43 |
| CaO | 25.84 | 0.11 | 19.31 | 0.53 | 24.45 | 0.20 | 44.79 | 0.27 |
| $Na_2O$ | 0.01 | 0.01 | 0.57 | 0.24 | 0.01 | 0.01 | 0.04 | 0.03 |
| Total | 99.93 | 0.20 | 100.09 | 0.49 | 99.61 | 0.25 | 98.90 | 0.58 |

*Cpx denotes clinopyroxenes coexisting with melt at T of the diffusion experiment; numbers in brackets denote number of measurements; std is the one-sigma standard deviation, avg. stands for average and Di. for diopside.*

***Table A3**: Cpx core and rim compositions of natural cpx phenocrysts (basaltic dyke, Adamello) determined by EPMA ("standard") and by EDS at the FEG-SEM.*

| | standard | | core (# 9) | | rim (# 9) | |
|---|---|---|---|---|---|---|
| wt. % | avg. (17) | std. | avg. (15) | std. | avg. (15) | std. |
| $SiO_2$ | 52.29 | 0.12 | 53.03 | 0.10 | 50.17 | 0.13 |
| $Al_2O_3$ | 2.63 | 0.06 | 2.81 | 0.03 | 5.83 | 0.02 |
| $Fe_2O_3$ | 5.67 | 0.39 | 6.07 | 0.10 | 6.35 | 0.09 |
| MgO | 16.40 | 0.12 | 16.66 | 0.05 | 14.72 | 0.05 |
| CaO | 22.34 | 0.36 | 22.54 | 0.07 | 23.22 | 0.08 |
| $Na_2O$ | 0.17 | 0.17 | 0.09 | 0.02 | 0.19 | 0.03 |
| Total | 99.50 | 0.13 | 101.20 | 0.24 | 100.49 | 0.26 |
| | | | | | | |
| $X_{Mg}$ ($Fe_{tot}$) | 0.852 | | 0.845 | | 0.821 | |
| Al | 0.115 | | 0.120 | | 0.252 | |

*Numbers in brackets denote the number of measurements; std. refers to the one-sigma standard deviation and avg. to the average; $X_{Mg}$ = (Mg/(Mg+Fe) and Al = Al-content in cations p.f.u.).*
